# Automated Aqueductal CSF Flow Analysis in Spontaneous Intracranial Hypotension: Hemodynamic Quantification and Exploratory Waveform Morphology Assessment Using Cine PC-MRI

**DOI:** 10.3390/diagnostics16121939

**Published:** 2026-06-22

**Authors:** Yi-Jhe Huang, Wen-Hsien Chen, Hung-Chieh Chen, Da-Chuan Cheng

**Affiliations:** 1 Graduate Institute of Biomedical Sciences, China Medical University, Taichung 404328, Taiwan; yijhe.huang@msa.hinet.net; 2 Department of Radiology, Taichung Veterans General Hospital, Taichung 407219, Taiwan; chenws.tw@gmail.com (W.-H.C.); hungchiehchen@gmail.com (H.-C.C.); 3 Department of Post-Baccalaureate Medicine, College of Medicine, National Chung Hsing University, Taichung 402202, Taiwan; 4 College of Medicine, National Yang Ming Chiao Tung University, Taipei 112304, Taiwan; 5 Department of Biomedical Imaging and Radiological Science, China Medical University, Taichung 404, Taiwan

**Keywords:** spontaneous intracranial hypotension (SIH), phase-contrast MRI (PC-MRI), deep learning, cerebrospinal fluid (CSF) dynamics, waveform morphology, AI-derived feature

## Abstract

**Background/Objectives:** Spontaneous intracranial hypotension (SIH) is caused by spinal cerebrospinal fluid (CSF) leakage and is typically diagnosed by clinical presentation and characteristic MRI signs; however, objective tools for monitoring physiological changes and treatment response remain limited. Cine phase-contrast MRI (PC-MRI) enables noninvasive quantification of aqueductal CSF dynamics, yet reliable analysis is challenging since the cerebral aqueduct is extremely small and susceptible to low contrast, partial volume effects, and ROI-dependent measurement variability—particularly in SIH where CSF pulsatility is often reduced. **Methods:** We propose an end-to-end automated framework that integrates (1) a cascade localization–segmentation strategy, consisting of Tiny YOLOv4 detection followed by MultiResUNet segmentation on a YOLOv4-derived cropped ROI; (2) physiology-informed pulsatility-based segmentation (PUBS) to refine anatomical masks into functional flow ROIs; and (3) one-dimensional convolutional neural networks (1D-CNNs) to extract exploratory waveform morphology features from 32-phase cardiac-cycle velocity waveforms. The study includes 39 participants, yielding 59 cine PC-MRI examinations: 11 controls, 28 Pre-treatment SIH scans and 20 Post-treatment Recovery scans. **Results:** The cascade model significantly improves segmentation robustness compared with a full-image baseline, achieving higher Dice scores and markedly lower boundary errors across cohorts (e.g., Pre-treatment SIH HD95: 1.66 ± 0.74 px vs. 15.37 ± 44.98 px). PUBS refinement reduces quantification deviation from expert manual references in SIH (mean relative error: 7.4% to 5.6%) and improves diagnostic performance for multiple hemodynamic parameters (e.g., downward mean flow AUC: 0.747 to 0.792). For waveform morphology analysis, the end-to-end 1D-CNN classifier was evaluated using repeated-seed participant-level grouped LOOCV. The repeated-seed ensemble prediction showed modest out-of-sample discrimination between Normal controls and Pre-treatment SIH scans, with an AUC of 0.646, a bootstrap 95% confidence interval of 0.455–0.826, and a permutation-test *p*-value of 0.072. Separately, exploratory analysis of the final baseline-trained 1D-CNN latent space showed marked, apparent Normal-versus-SIH separability and an intermediate recovery distribution in PCA space, suggesting that aqueductal waveform morphology may encode SIH-related physiological information. **Conclusions:** These findings suggest that SIH-related information may be reflected not only in flow magnitude but also in aqueductal CSF waveform morphology. However, the modest and statistically non-significant out-of-sample performance of the end-to-end 1D-CNN classifier indicates that morphology-based AI features should currently be regarded as exploratory biomarker candidates rather than validated stand-alone diagnostic tools. Larger independent cohorts are required to confirm their reproducibility, physiological meaning, and clinical utility.

## 1. Introduction

Spontaneous intracranial hypotension (SIH) is a potentially debilitating disorder caused by cerebrospinal fluid (CSF) leakage from the spinal thecal sac. This leakage reduces intracranial CSF volume and pressure [1]. Clinically, SIH is characterized by orthostatic headache and typical magnetic resonance imaging (MRI) findings. These findings include pachymeningeal enhancement, pituitary hyperemia, venous engorgement, and brain sagging [2,3]. Epidural blood patching (EBP) is a standard treatment for SIH. However, routine clinical practice still lacks reliable and objective tools to monitor treatment response and to characterize the underlying physiological changes [4,5].

Evidence suggests that SIH is not only a disorder of CSF volume loss. It also involves altered craniospinal biomechanics and compliance. When intracranial CSF volume decreases, compensatory mechanisms change the intracranial pressure–volume relationship. This change can alter intracranial elastance and craniospinal coupling [6,7,8,9]. These biomechanical changes are expected to affect CSF flow dynamics across the craniospinal axis, including the cervical spine [10], and especially in the cerebral aqueduct. The aqueduct is a narrow conduit and may be sensitive to small changes in intracranial compliance. Computational and mechanistic models also suggest that brain deformability and fluid–structure interaction (FSI) can shape aqueductal CSF waveforms. These effects include bidirectional flow components that may be missed by rigid-wall assumptions [11]. Therefore, aqueductal CSF flow waveforms may contain physiological information beyond conventional scalar measures.

Phase-contrast MRI (PC-MRI) is a noninvasive clinical tool for quantifying CSF flow dynamics. In current practice, clinicians often define aqueduct regions of interest (ROIs) manually using magnitude images. They then report scalar parameters, such as peak velocity or stroke volume, which may be underestimated when ROI placement is affected by boundary errors [12,13,14,15]. Accurate aqueduct quantification remains difficult. The aqueduct occupies only a tiny fraction of the image and is sensitive to partial-volume effects, phase noise, and physiological motion. Net-flow measurements can also be confounded by respiratory influences [15,16,17]. These problems are more pronounced in SIH. Aqueductal CSF velocities are often lower than in conditions with higher pulsatility. This reduction weakens flow-related contrast and increases uncertainty in ROI delineation [12,13,14].

Early semiautomatic approaches showed that anatomically plausible ROIs may not produce physiologically reliable measurements. This issue is critical for peak-related metrics, which are sensitive to ROI boundaries and partial-volume effects [17]. To address this, physiology-informed methods have been proposed. For example, pulsatility-based segmentation (PUBS) uses temporal consistency across the cardiac cycle to identify functional flow regions rather than relying only on anatomical intensity [18]. However, most pipelines still focus on spatial segmentation. They then compress the waveform into a small set of scalar metrics, leaving much of its temporal information underutilized.

Deep learning has improved automation in medical image analysis [19]. Object detection models such as YOLO can localize small targets in complex anatomical backgrounds [20,21]. Segmentation networks such as U-Net and its variants (including MultiResUNet) can delineate small structures using multi-scale features [22,23]. Deep learning has also been used for automated aqueduct CSF flow analysis [24]. In parallel, one-dimensional convolutional neural networks (1D-CNNs) can extract morphological features from biomedical time-series signals. This enables waveform characterization beyond point-based measurements [25,26,27]. Although AI-assisted quantitative PC-MRI has recently shown promise for assessing cervical CSF flow in SIH [10], aqueductal CSF flow analysis presents distinct technical challenges because of the extremely small size of the cerebral aqueduct, low flow-related contrast, and ROI-dependent measurement variability. To our knowledge, an integrated framework combining spatial localization, physiology-informed refinement, and waveform-morphology learning has not yet been explored for aqueductal CSF flow analysis in SIH.

In this study, we develop a fully automated end-to-end framework to quantify aqueductal CSF flow dynamics in SIH using cine PC-MRI. Our method includes:

(1) cascade localization and segmentation to delineate the aqueduct in low-contrast images,

(2) pulsatility-based refinement to obtain physiologically meaningful flow ROIs, and

(3) 1D-CNN–based waveform analysis to extract morphology-related features beyond conventional scalar metrics.

By combining spatial accuracy with temporal waveform analysis, our framework aims to improve the reproducibility of aqueductal CSF flow quantification and to explore waveform-based signatures that may support longitudinal assessment.

## 2. Materials and Methods

### 2.1. Study Population

A total of 39 participants were included in this retrospective study, yielding 59 cine PC-MRI examinations. The examinations were analyzed in three groups: (1) Normal control scans (*N* = 11), (2) Pre-treatment SIH scans (*N* = 28), and (3) Post-treatment Recovery scans (*N* = 20), which were obtained from patients in the Pre-treatment SIH cohort after epidural blood patching and clinical/radiological recovery on follow-up imaging. The study was approved by the Institutional Review Board of Taichung Veterans General Hospital (IRB-CE22242B).

### 2.2. Image Acquisition

Phase-contrast MRI (PC-MRI) was performed using a 1.5T scanner (MAGNETOM Aera, Siemens Healthcare, Erlangen, Germany). The scan plane was positioned perpendicular to the aqueduct of Sylvius at the level of the midbrain. Imaging parameters were as follows: velocity encoding (venc) = 20 cm/s; repetition time (TR) = 134 ms; echo time (TE) = 10 ms; flip angle = 10°; field of view (FOV) = 140 mm; matrix size = 384 × 384 pixels; and slice thickness = 5 mm. A total of 32 cardiac phases were reconstructed retrospectively using electrocardiogram (ECG) gating to cover the entire cardiac cycle.

### 2.3. Cascade Deep Learning Framework

A cascade “localization-then-segmentation” framework was implemented to segment the cerebral aqueduct from low-contrast magnitude images, especially in SIH. The cascade approach was compared with a direct full-image segmentation model. The computational pipeline and statistical analyses were implemented using MATLAB R2025a (The MathWorks, Inc., Natick, MA, USA) [28].

#### 2.3.1. Stage 1: Midbrain Localization Model

**Figure 1:**
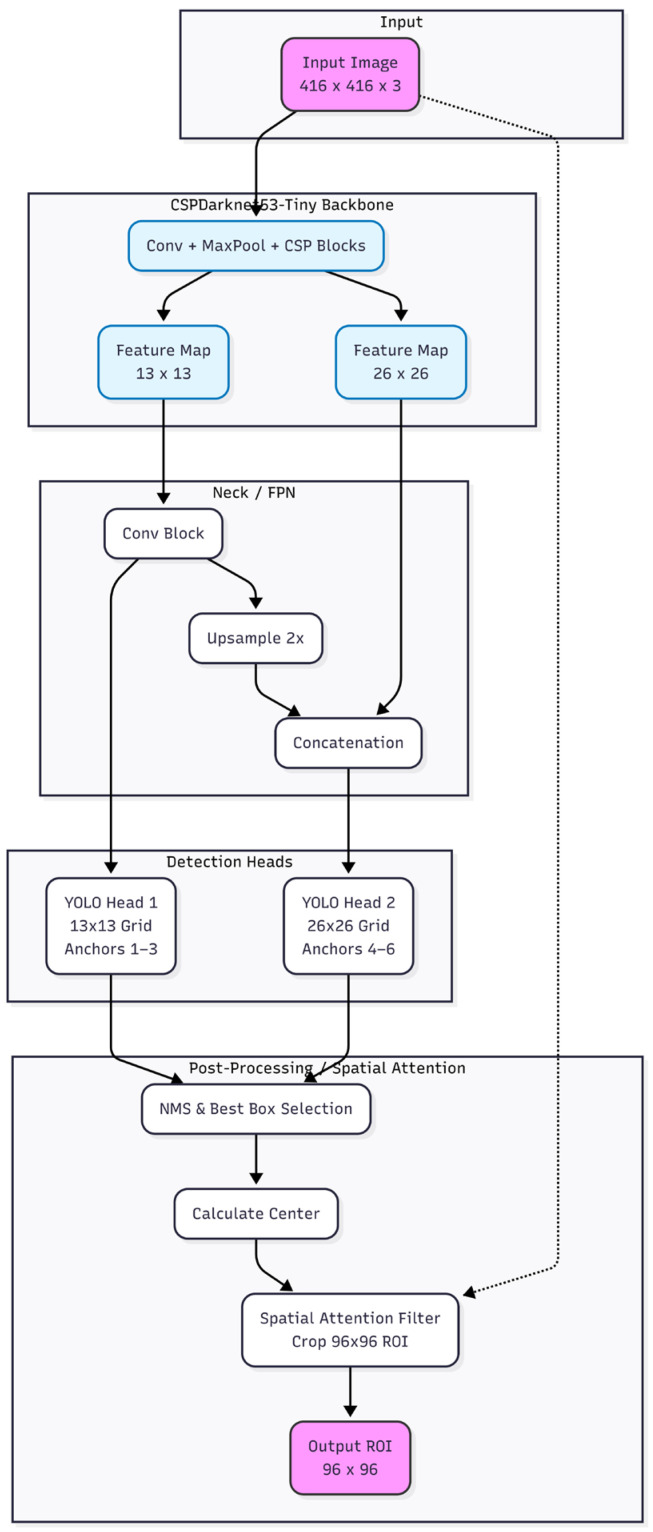
Architecture of the Tiny YOLOv4 network used for midbrain localization. The model consists of a CSPDarknet53-tiny backbone and a feature pyramid network for multi-scale detection, based on the YOLOv4/Scaled-YOLOv4 framework [20,21].

Midbrain localization was performed using a Tiny YOLOv4 object detector [21] (Figure 1). To ensure robust feature learning independent of the clinical evaluation cohort, midbrain localization was trained using a dedicated dataset of 100 independent subjects. This localization dataset had no subject-level or scan-level overlap with either the MultiResUNet segmentation dataset or the clinical analysis cohort. Each participant contributed exactly one representative static magnitude image (total *N* = 100 images). The dataset was randomly partitioned into training (70%, *N* = 70), validation (15%, *N* = 15), and testing (15%, *N* = 15) sets. Data augmentation was applied exclusively during training. Anchor boxes were estimated using an IoU-based distance metric to cluster ground-truth boxes into six anchors. The network was trained for 120 epochs using the Adam optimizer (mini-batch size = 8) with L2 regularization of 5 × 10^−4^. The input resolution was 416 × 416 pixels. For the object detection component, the model optimized a multi-part loss function rather than simple epoch-by-epoch classification accuracy. The training and validation loss curves demonstrated rapid and stable convergence without obvious divergence between the two curves (Supplementary Figure S1). Furthermore, when evaluated on the independent test set, the model achieved an Average Precision (AP) of 86.67%. The precision–recall analysis showed that precision remained 1.000 up to a recall of 0.867, indicating no false-positive localizations within this operating range (Supplementary Figure S2). During inference, the predicted midbrain bounding box defined the center for cropping a fixed 96 × 96-pixel ROI. This cropping reduced background noise and standardized the input for downstream segmentation.

#### 2.3.2. Stage 2: Deep Learning Segmentation Strategies

**Figure 2:**
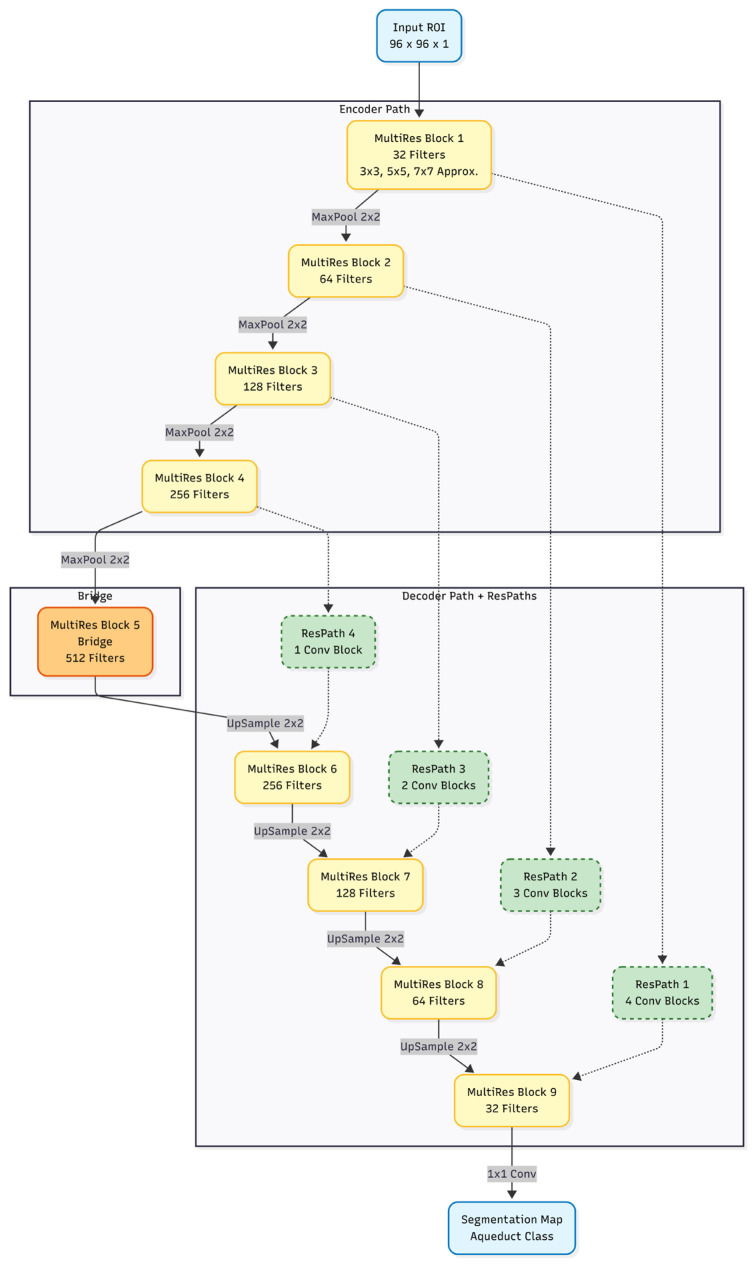
Proposed MultiResUNet architecture. The illustrated model processes a cropped 96 × 96 ROI. It uses MultiRes Blocks (yellow) to extract multi-scale spatial features through cascaded convolutional operations that approximate multiple receptive fields, and ResPaths (green) to reduce the semantic gap between encoder and decoder features. The comparative baseline model used the same network architecture but operated on 384 × 384 full-field inputs, with the mini-batch size reduced to 4 to accommodate memory constraints.

Two segmentation strategies based on MultiResUNet [22] were implemented (Figure 2). MultiResUNet was selected for multi-scale feature extraction using MultiRes blocks and for bridging encoder–decoder features using ResPaths.

(1) Proposed Cascade Strategy (Cropped Input):

To capture sufficient anatomical variance, this model was trained on a separate, distinct dataset of 100 independent subjects (one static image per subject, total *N* = 100 images), which had no overlap with the YOLO localization dataset or the clinical analysis cohort. The dataset was split using an 80/20 hold-out approach, allocating 80% for training (*N* = 80) and 20% for validation (*N* = 20) to monitor training behavior and validation loss. Furthermore, the segmentation network was trained on the 96 × 96-pixel ROI produced by YOLO. This design reduced class imbalance by focusing on the target region. A weighted pixel-wise cross-entropy loss was used with a fixed penalty weight (weight factor = 1.5) for the aqueduct class. Training used Adam (learning rate = 10^−4^), mini-batch size = 16, and augmentation (rotation ±15°, translation ±10 pixels) for 200 epochs. To evaluate the training dynamics of the segmentation network, both training and validation loss, alongside segmentation accuracy, were monitored across the training epochs. The validation curves closely tracked the training curves throughout the 200-epoch training process, suggesting stable learning behavior without obvious overfitting (Supplementary Figure S3).

(2) Baseline Full-Image Strategy:

For comparison, a baseline MultiResUNet model was trained on full-field images (384 × 384 pixels) without prior localization. The core optimizer and augmentation strategy were kept consistent with the cascade model, whereas the mini-batch size, number of epochs, and class-weighting strategy were adjusted to accommodate the full-field input setting. For the full-image baseline, the mini-batch size was reduced to 4 because of the higher memory demand of the 384 × 384 input, and the training duration was set to 50 epochs. Because the full-image model used one-fourth of the mini-batch size used by the cropped cascade model, this design yielded a comparable number of gradient-update iterations under a similar computational budget. In full-field scans, aqueduct pixels were extremely sparse and occupied <0.1% of the image area. To handle this class imbalance, median frequency balancing was used to compute class weights dynamically. This approach differed from the cascade model, which used a fixed class weight. Dynamic weighting was needed to prevent the background class from dominating the gradients during training.

After training, both the cascade model and the full-image baseline model were evaluated on the clinical analysis cohort, which was not used for model training or validation. Segmentation performance was assessed separately in Normal controls, Pre-treatment SIH patients, and Post-treatment Recovery scans using expert manual annotations as the reference standard.

### 2.4. Physiological Refinement: Pulsatility-Based Segmentation (PUBS)

An anatomical mask could include noise and partial-volume pixels. Therefore, the PUBS algorithm [18] was applied to derive a functional flow ROI. First, a representative reference velocity waveform was obtained from the raw mask using a clustering approach. Next, a pixel-wise correlation map was computed by measuring temporal similarity between each pixel waveform (within a dilated mask) and the reference waveform. The cross-correlation coefficient ($P_{xy}$) is defined in Equation (1) [18]. The reference waveform and each pixel waveform were compared across the cardiac cycle (*N* = 32 phases), after subtracting their temporal means.

(1) $$P_{xy}=\frac{\sum_{k=1}^{N}\left(R_{k}-\bar{R}\right)\left(V_{xy,k}-{\bar{V}}_{xy}\right)}{\sqrt{\sum_{k=1}^{N}{\left(R_{k}-\bar{R}\right)}^{2}\sum_{k=1}^{N}{\left(V_{xy,k}-{\bar{V}}_{xy}\right)}^{2}}}$$

where $R_{k}$ represents the velocity waveform of the reference and $V_{xy,k}$ represents the velocity at location (x,y) at the k−th cardiac phase. $\bar{R}$ and ${\bar{V}}_{xy}$ denote their respective temporal means over the cardiac cycle (*N* = 32 phases).

To identify the most stable region for flow quantification, an automated plateau identification algorithm was implemented to determine the optimal correlation threshold (${Th}^{*}$). The fundamental assumption was that within the true lumen, the calculated flow should exhibit minimal sensitivity to slight variations in the threshold, thereby mitigating partial volume effects at the boundaries. Mean flow rates were calculated across a predefined threshold range (Th = 0.50 to 0.84, increment = $\frac{∆Th}{5}$). To identify the region least sensitive to threshold variations, a sliding window (∆Th = 0.10) was applied to compute the local coefficient of variation (CV) along the flow curve. The CV at each candidate threshold ${Th}_{i}$ was computed as:

(2) $$CV\left({Th}_{i}\right)=\;\frac{\sigma_{flow({Th}_{i})}}{\left|\mu_{flow({Th}_{i})}\right|}{|}_{\Omega },\;where\;\Omega \;=\left\{{Th}_{i}-2\frac{∆Th}{5},\;{Th}_{i}+2\frac{∆Th}{5}\right\},\;{Th}_{i}\in [0.50,\;0.84]$$

where $\sigma_{flow(Th)}$ and $\mu_{flow(Th)}$ denote the standard deviation and the mean of the flow rates within the specified window, respectively. The optimal threshold (${Th}^{*}$) was then selected as the threshold yielding the minimum CV:

(3) $${Th}^{*}=arg\underset{{\mathrm{Th}}_{i}}{\min}CV({Th}_{i})$$

The rationale for computationally defining this plateau by minimizing the local CV of the calculated flow rather than merely relying on geometric pixel count was physiologically motivated. Calculating the plateau based on flow (Area × Velocity) inherently assigned lower mathematical weights to noisy low-velocity peripheral pixels that were subject to partial volume effects while simultaneously emphasizing the high-velocity core luminal signal. This ensured that the optimal threshold was selected at the point of maximum functional stability rather than mere spatial consistency.

Once the optimal threshold (${Th}^{*}$) was determined, it was applied to the pixel-wise cross-correlation coefficient map. Pixels with a correlation value ($P_{xy})$ higher than this optimal threshold were kept. These pixels formed the functional region of interest (ROI). The ROI was then used for subsequent flow quantification.

### 2.5. Background Correction

Prior to flow analysis, phase offset errors caused by eddy currents were corrected using a local background correction method [29,30]. We specifically targeted the midbrain region for this correction because phase offset errors were spatially inhomogeneous. Static tissue near the aqueduct provided a more accurate estimate of the local phase baseline. This estimate was more reliable than one derived from distant brain regions.

**Figure 3:**
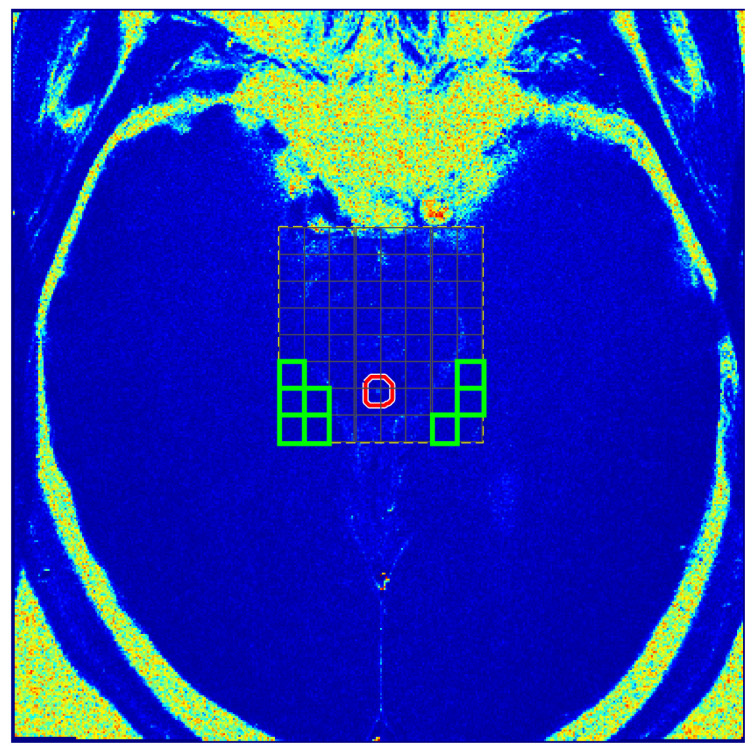
Automated local background correction strategy. The midbrain region is divided into an 8 × 8 grid to identify static tissue. The red contour delineates the exclusion zone (dilated from the aqueduct) to avoid periaqueductal pulsatility artifacts. The green rectangles highlight the final selected background ROIs, which are characterized by the lowest temporal standard deviation values outside the exclusion zone and are used for velocity offset correction.

An automated algorithm partitioned the midbrain region into an 8 × 8 grid. To prevent signal contamination from the high-velocity CSF flow, an “exclusion zone” was established by dilating the aqueduct ROI with a disk structuring element (radius = 6 pixels). Grid cells overlapping with this exclusion zone or containing insufficient tissue pixels were automatically excluded. From the remaining candidates, the algorithm identified the optimal “static” tissue by selecting the top 8 grid cells with the lowest temporal standard deviation (t-SD) of phase velocity. The automated background selection procedure is illustrated in Figure 3.

The t-SD for each grid ($\sigma_{t,g}$) was calculated as:

(4) $$\sigma_{t,g}=\sqrt{\frac{1}{N-1}\sum_{k=1}^{N}{\left(v_{g}\left(t_{k}\right)-{\bar{v}}_{g}\right)}^{2}}$$

where N denotes the number of cardiac phases (*N* = 32), $v_{g}\left(t_{k}\right)$ denotes the mean velocity of the grid at phase k, and ${\bar{v}}_{g}$ is the temporal average velocity over the cardiac cycle. This metric effectively differentiated static tissue (low t-SD) from pulsatile vascular structures (high t-SD). The mean phase velocity across these selected stable regions was calculated for each time frame and subtracted from the aqueduct velocity. This mechanism was designed to provide stable baseline correction even in the presence of surrounding physiological motion.

### 2.6. Hemodynamic Parameter Calculation

Based on the refined functional ROI, a comprehensive set of hemodynamic parameters was calculated for each examination. These parameters were categorized into volumetric, flow rate, and velocity metrics [12,13,14,15]:

(1) Stroke Volume Metrics (mL/cycle):
  - Upward CSF total flow: Integral of flow during the diastolic (cranial) phase (the volume of CSF returning towards the third ventricle).
  - Downward CSF total flow: Integral of flow during the systolic (caudal) phase (the volume of CSF displaced towards the spinal canal).
  - Absolute stroke volume: Sum of the absolute upward and downward volumes.
(2) Flow Rate Metrics (mL/s):
  - Upward Mean Flow: Calculated for the upward (cranial) phases.
  - Downward Mean Flow: Calculated for the downward (caudal) phases.
  - Total Mean Flow: Calculated as the sum of absolute Upward and Downward Mean Flow to assess the overall magnitude and pulsatile intensity of the CSF.
  - Upward Peak Flow: The maximum instantaneous flow rate recorded during the upward phases.
  - Downward Peak Flow: The most negative instantaneous flow rate recorded during the downward phases.
  - Summation of Peak Flow: Calculated by adding the absolute Upward and Downward Peak Flow, also derived to characterize the peak-to-peak flow amplitude.
(3) Velocity Metrics (cm/s):
  - Upward Peak Velocities: The maximum pixel velocity identified within the ROI during the upward (diastolic) phases.
  - Downward Peak Velocities: The most negative pixel velocity identified within the ROI during the downward (systolic) phases.

### 2.7. Waveform Morphology Analysis Using 1D-CNN

#### 2.7.1. Input Data Extraction and Preprocessing

Since the peak velocity of aqueduct flow is an effective feature for distinguishing between Normal controls and SIH patients [13], we used a method to extract the top 5% peak velocity of each examination. To ensure the robustness of the extracted features and minimize single-pixel noise, the process was as follows:

Let $V(\overset{⃑}{x},\;t)$ denote the velocity at a spatial coordinate $\overset{⃑}{x}$ within the binary mask M from the PUBS-defined ROI, at a given time point t in the cardiac cycle. The set of all velocity values within the ROI at time t was defined as:

(5) $$S_{t}\;=\;\{V(\overset{⃑}{x},\;t)\;|\overset{⃑}{x}\;\in M\}$$

To identify the top 5% highest magnitudes, we first defined the $95^{th}$ percentile threshold $P_{95}$ based on the absolute values of the pixels in $S_{t}$:

(6) $$P_{95}(t)\;=\;percentile(\{|v|\;:\;v\;{\in S}_{t}\},\;95)$$

We then defined the subset of “strong pixels” ${S\prime }_{t}$, which contained the original velocity values whose magnitudes were greater than or equal to this threshold:

(7) $${S^{\prime }}_{t}=\;\{v\;\in S_{t}\;\left|\;\left|v\right|\right.\geq P_{95}(t)\}$$

Finally, the peak velocity curve at time t, denoted as $C_{peak}(t)$, is the arithmetic mean of the values in ${S^{\prime }}_{t}$:

(8) $$C_{peak}\left(t\right)=\left\{\begin{array}{cc} \frac{1}{\left|{S^{\prime }}_{t}\right|}\sum_{v\;\in {S^{\prime }}_{t}}v, & \;if\;S_{t}\neq \emptyset \\ 0, & \;if\;S_{t}=\emptyset \;(\mathrm{empty}\;\mathrm{set}) \end{array}\right.$$

#### 2.7.2. Normalization and Hybrid Feature Strategy

Subsequently, the extracted 32-point temporal waveform ($C_{peak}(t))$ was used to derive two complementary categories of measurements:

(1) Hemodynamic Parameter: Physiologically, the peak flow of CSF reflects the dominant systolic component of CSF pulsation. To capture this dynamic, we utilized the raw waveform—preserving its original physical units—to quantify the true flow magnitude. Specifically, the maximum absolute CSF velocity across the entire cardiac cycle was extracted and reported as PSV (cm/s), representing the peak CSF velocity magnitude used in the present analysis.
(2) Morphological Input for AI: To encourage the deep learning model to focus on morphology-related variations—such as systolic sharpness, decay rate, and diastolic rebounds—it was necessary to isolate intrinsic waveform shape patterns from the individual flow magnitude. To achieve this, the entire 32-point raw waveform underwent Z-score normalization prior to model input. The normalized value $z_{t}$ at each time point t was calculated as follows:

(9) $$z_{t}=\frac{x_{t}-\mu }{\sigma }$$

where $x_{t}$ represents the raw velocity at time point t, with μ and σ denoting the temporal mean and standard deviation of the waveform across the cardiac cycle, respectively.

This design, combining top 5% peak-velocity extraction with waveform-wise Z-score normalization, was intended to reduce the influence of minor threshold-dependent boundary variations on downstream morphology learning. By emphasizing high-magnitude luminal velocity signals and reducing absolute amplitude dominance, this preprocessing strategy provided a more consistent waveform input for the 1D-CNN.

#### 2.7.3. 1D-CNN Architecture

**Figure 4:**
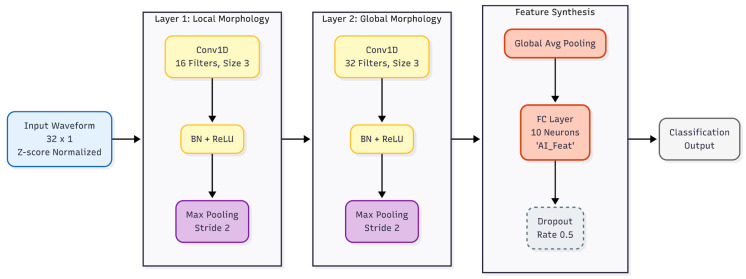
Schematic of the 1D-CNN waveform feature extraction network. The model transforms normalized 32-point CSF velocity waveforms into compact latent representations. Hierarchical convolutional layers detect local temporal patterns, and global average pooling summarizes the feature maps before generating 10 latent features.

A custom 1D Convolutional Neural Network (1D-CNN) [27] was designed to learn morphology-related representations from normalized 32-point CSF velocity waveforms. The network architecture consisted of two sequential convolutional blocks followed by a feature synthesis head (Figure 4):

(1) Morphological Detection Blocks: The network comprised two convolutional blocks. The first block utilized 16 filters (kernel size: 3) and the second used 32 filters (kernel size: 3). Crucially, each convolutional layer was immediately followed by a Batch Normalization layer to stabilize gradient propagation and a ReLU activation function.
(2) Feature Condensation: Max-pooling layers (stride 2) were placed after each block to downsample the feature maps, preserving the most salient activations while reducing dimensionality.
(3) Latent Feature Synthesis: A Global Average Pooling (GAP) layer was then used to aggregate temporal feature maps into a compact representation. The resulting vector was passed to a fully connected layer with 10 neurons, generating 10 latent waveform features, designated AI_Feat_1 to AI_Feat_10. A final fully connected layer and softmax layer were used for binary classification between Normal controls and Pre-treatment SIH scans.

The 10-neuron latent feature layer was used only for exploratory representation analysis after the primary classifier validation. These latent features were not interpreted as independently validated diagnostic biomarkers.

#### 2.7.4. Repeated-Seed Participant-Level Grouped LOOCV Classifier Validation

The primary diagnostic validation of the end-to-end 1D-CNN classifier was performed under a binary Normal-versus-Pre-treatment SIH setting. Post-treatment Recovery scans were excluded from classifier training and testing and were reserved for subsequent exploratory projection analysis.

To minimize subject-level leakage, a participant-level grouped leave-one-out cross-validation (LOOCV) strategy was used. In each fold, all data belonging to one participant were held out as the test set, while the remaining participants were used for training. The 32 cardiac phases of each waveform were treated as a single sequence and were never split into independent samples.

Because CNN training can be sensitive to random initialization, mini-batch ordering, and augmentation sampling in small datasets, the grouped LOOCV experiment was repeated across 30 random seeds. For each seed and each held-out participant, the out-of-sample softmax probability for the SIH class was recorded. The final ensemble SIH probability for each participant was calculated by averaging the out-of-sample probabilities across all 30 seeds. This repeated-seed ensemble probability was used as the primary classifier-level performance estimate. No single best-performing seed was selected for reporting.

Data augmentation was applied only within the training fold. Augmentation included random circular temporal shifting within ±2 cardiac phases and additive zero-mean Gaussian noise scaled to 5% of the normalized waveform standard deviation. The held-out participant remained completely unseen and unaugmented during training. The 1D-CNN was trained using the Adam optimizer, a mini-batch size of 64, an initial learning rate of 0.001, L2 regularization of 0.01, and 50 epochs for each LOOCV fold.

#### 2.7.5. Final Baseline-Trained Latent Feature Extraction and Recovery Projection

After completing the repeated-seed grouped LOOCV classifier validation, a final baseline-trained 1D-CNN was trained using the complete baseline diagnostic cohort, including Normal controls and Pre-treatment SIH scans only. This final model was not used to estimate independent out-of-sample classifier performance. Instead, it was used as a fixed representation-learning model for exploratory latent feature extraction and recovery projection.

The numerical activations from the 10-neuron latent feature layer were extracted as AI_Feat_1 to AI_Feat_10 for Normal, Pre-treatment SIH, and Post-treatment Recovery scans. Recovery scans were passed through the fixed final baseline-trained model but were not used to train the binary classifier. The final baseline-trained model showed stable training convergence, as demonstrated by decreasing training loss and increasing training accuracy over 80 epochs in Supplementary Figure S4. This model was subsequently used for exploratory latent feature extraction, PCA projection, and recovery projection.

Principal component analysis (PCA) was performed using only the baseline diagnostic cohort after Z-score standardization of AI_Feat_1 to AI_Feat_10. Post-treatment Recovery scans were then projected into the same PCA space without influencing the PCA axes. PCA loadings were calculated to describe the contribution of each latent feature to PC1 and PC2, and recovery scans were visualized relative to the morphology space defined by Normal controls and Pre-treatment SIH scans.

### 2.8. Statistical Analysis

All statistical analyses and data processing were performed using MATLAB R2025a. Continuous variables are presented as Mean ± Standard Deviation (SD), and categorical variables are expressed as counts. A two-sided p-value of less than 0.05 was considered statistically significant.

#### 2.8.1. Clinical Demographics and Segmentation Performance

For demographic comparisons, differences in age were evaluated using Student’s t-test, while sex distribution differences were assessed using Fisher’s exact test.

To validate the segmentation performance, the improvement of the proposed cascade model over the full-image baseline was evaluated using the non-parametric Wilcoxon Signed-Rank Test for paired comparisons of the Dice Similarity Coefficient (DSC) and 95% Hausdorff Distance (HD95).

#### 2.8.2. Hemodynamic Parameter Analysis

Comparisons of hemodynamic parameters (e.g., flow rates, velocities, stroke volume) between Normal controls, Pre-treatment SIH, and Post-treatment Recovery groups were conducted using the Mann–Whitney U test for independent samples. To quantify the diagnostic performance of these parameters in distinguishing SIH patients from controls, Receiver Operating Characteristic (ROC) curve analysis was performed, and the Area Under the Curve (AUC) was calculated. Additionally, radar charts were utilized to visualize the comprehensive diagnostic efficacy across different methods (Manual, Cascade-only, and Cascade + PUBS).

#### 2.8.3. Deep Learning Feature Analysis and Diagnostic Validation

For waveform morphology analysis, the primary diagnostic validation was performed using the repeated-seed participant-level grouped LOOCV predictions of the end-to-end 1D-CNN classifier. In each LOOCV fold, the SIH-class softmax probability of the held-out participant was recorded as a strictly out-of-sample prediction. To account for stochastic variability in CNN training, this grouped LOOCV procedure was repeated across 30 random seeds. For each participant, the ensemble SIH probability was calculated by averaging the out-of-sample probabilities across all repeated-seed runs. The final ROC curve and AUC of the end-to-end 1D-CNN classifier were generated using these ensemble probabilities. Sensitivity, specificity, accuracy, and balanced accuracy were calculated at the optimal threshold determined by Youden’s Index.

To quantify uncertainty in the primary classifier-level AUC, bootstrap resampling was performed to estimate the 95% confidence interval. A permutation test was also conducted by randomly permuting the diagnostic labels and recalculating the ensemble AUC to evaluate whether the observed classifier performance exceeded chance-level discrimination. No single best-performing seed was selected for reporting; the repeated-seed ensemble result was used as the primary classifier-level validation.

After the primary LOOCV, exploratory latent-space analyses were performed using the final baseline-trained 1D-CNN feature extractor. The 10 AI-derived latent features, AI_Feat_1 to AI_Feat_10, were extracted from the final 10-neuron feature layer for Normal controls, Pre-treatment SIH scans, and Post-treatment Recovery scans. Group-wise distributions of each latent feature were summarized as mean ± SD and visualized using box-and-swarm plots. Pairwise group comparisons among Normal, SIH, and Recovery groups were performed using the Mann–Whitney U test. These feature-level analyses were interpreted as exploratory latent-space analyses rather than independent diagnostic validation because the features were extracted from a final model trained on the complete baseline diagnostic cohort.

Principal component analysis was performed on AI_Feat_1 to AI_Feat_10 extracted from the final baseline-trained 1D-CNN feature extractor. PCA fitting was performed using only the baseline diagnostic cohort after Z-score standardization. Post-treatment Recovery scans were then projected into this baseline-defined PCA space without influencing the PCA axes. This design allowed visualization of Recovery scans relative to the baseline-defined waveform morphology space. PCA loadings were calculated to describe the contribution of each latent feature to PC1 and PC2.

In addition to the primary repeated-seed grouped LOOCV, exploratory apparent ROC analyses were performed for PSV, final baseline-trained latent features, and the final baseline-trained 1D-CNN softmax probability. PSV was included as a conventional scalar velocity reference. The latent-feature model was based on AI_Feat_1 to AI_Feat_10, and the final 1D-CNN probability represented the SIH-class softmax output from the same final baseline-trained model. Because the latent-feature and final-model probability analyses were derived from a model trained on the complete baseline diagnostic cohort, these ROC analyses were interpreted as apparent separability rather than independent out-of-sample validation.

#### 2.8.4. Inter-Rater Reliability Analysis

To evaluate the reproducibility and objectivity of the manual reference standard, an inter-rater reliability analysis was performed on the entire dataset (*N* = 59). A second independent expert reader, blinded to the clinical status and group allocations of the participants, manually segmented the cerebral aqueduct. The spatial overlap and boundary alignment between the primary and secondary readers were quantitatively assessed using the Dice Similarity Coefficient (DSC), Intersection over Union (IoU), and the 95th percentile Hausdorff Distance (HD95).

## 3. Results

### 3.1. Demographic Characteristics of the Study Population

A total of 39 participants were included in the study. The cohort consisted of 11 Normal controls (Mean Age: 32.9 ± 5.3 years; 6 males/5 females) and 28 patients with spontaneous intracranial hypotension (SIH) (Mean Age: 40.1 ± 10.4 years; 7 males/21 females). Among the SIH group, 20 patients who exhibited clinical improvement following Epidural Blood Patch (EBP) treatment were included in the Post-treatment Recovery group subset.

Statistical analysis revealed a significant difference in age between the Normal controls and the Pre-treatment SIH patients (*p* = 0.036), with the SIH group being slightly older. However, there was no statistically significant difference in sex distribution (*p* = 0.13) between the two groups. Detailed demographic characteristics are summarized in Table 1.

### 3.2. Segmentation Performance and Ablation Study

To validate the efficacy of the proposed cascade architecture (YOLO localization + MultiResUNet segmentation), an ablation study was conducted by comparing it against a baseline MultiResUNet model trained on full-field images. Performance was evaluated using the Dice Similarity Coefficient (DSC), where a value closer to 1 indicates superior volumetric overlap, and the 95% Hausdorff Distance (HD95), where a lower value signifies higher boundary delineation accuracy. Table 2 summarizes the quantitative metrics across the three clinical cohorts. The proposed cascade framework consistently outperformed the full-image baseline in all groups.

**Figure 5:**
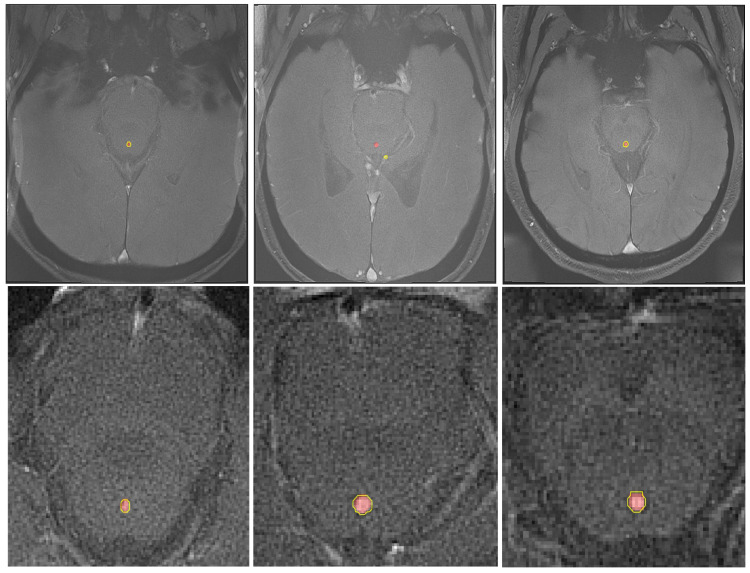
Visual assessment of segmentation performance. Top rows: Full-Image baseline; Bottom rows: Proposed cascade model. Red: Ground Truth; Yellow: Predicted Segmentation. The proposed method showed superior robustness, successfully localizing the target even in cases where the baseline model generated false positives (e.g., top-center panel).

Qualitative visual comparisons between the baseline full-image model and the proposed cascade architecture are presented in Figure 5. In these visualizations, the manual ground truth is overlaid in solid red, while the model’s predicted boundaries are outlined in yellow. Visual inspection revealed that the cascade model (bottom rows) consistently achieved superior segmentation performance, with predicted contours closely aligning with the ground truth across varying anatomical structures.

In contrast, the full-image model (top rows) exhibited significant instability in localizing small targets. A representative failure is shown in the center image of the top row, where the baseline model completely missed the true anatomical target and incorrectly predicted a false-positive region far from the ground truth.

Furthermore, the evaluation of inter-rater reliability between the two independent expert readers demonstrated near-perfect agreement across all clinical cohorts (Table 3). The average DSC exceeded 0.98 and the average IoU exceeded 0.97 across all groups. The mean HD95 values were all below 0.1 mm, indicating excellent boundary agreement between the two independent expert readers. These results support the high reproducibility of the annotation protocol and reduce the likelihood of major observer-dependent bias. Therefore, the segmentations delineated by the primary reader were considered suitable reference masks for downstream hemodynamic parameter extraction.

### 3.3. Impact of Physiological Signal Refinement (PUBS)

**Figure 6:**
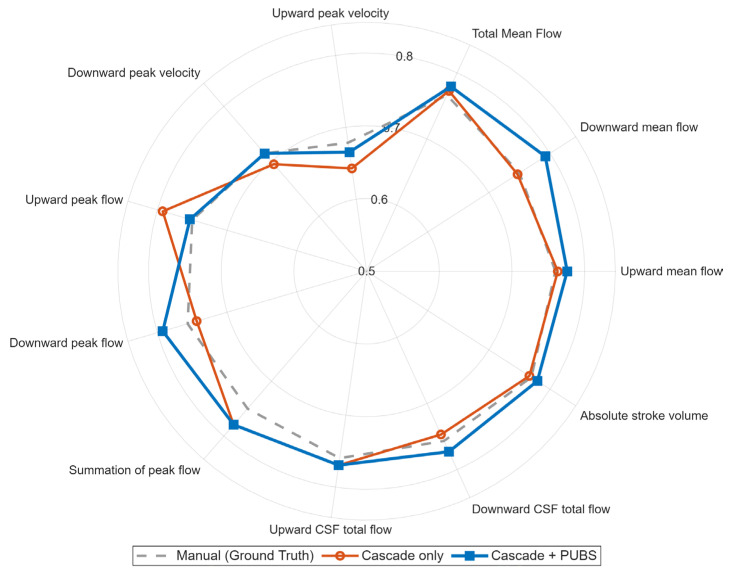
Radar chart comparison of diagnostic efficacy. The plot displays the AUC values (Normal vs. SIH) for hemodynamic parameters calculated by the Manual method (gray), the Cascade-only method (orange), and the proposed PUBS method (blue). The PUBS-refined framework showed improved overall diagnostic separability across most flow-derived parameters, particularly downward mean flow and downward peak flow. However, the magnitude of improvement varied across individual parameters.

**Figure 7:**
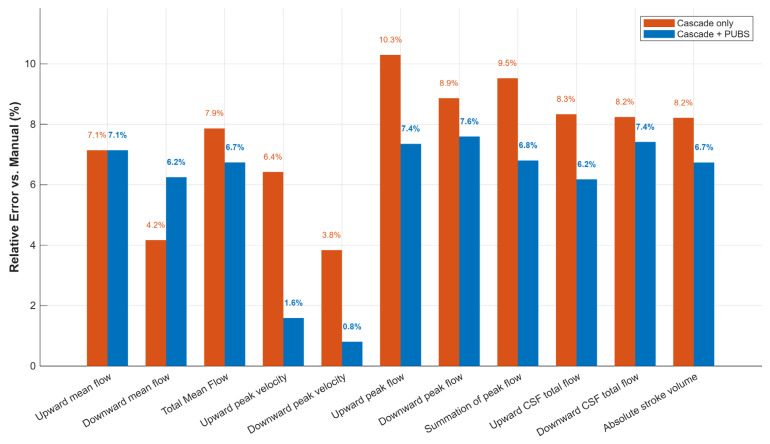
Comparison of measurement relative error. The chart displays the absolute percentage deviation of the Cascade-only method (orange) and the PUBS-refined method (blue) from the expert manual reference within the SIH cohort. The PUBS-refined framework showed a lower overall average relative error, indicating improved agreement with the manual reference standard. This improvement suggests that physiological signal refinement can reduce boundary-related deviations associated with anatomical-only segmentation.

To evaluate the contribution of the Pulsatility-Based Segmentation (PUBS) algorithm in refining hemodynamic assessment, we compared the flow metrics derived from the initial anatomical segmentation (Cascade-only) against those refined by PUBS (Cascade + PUBS). The comparative results are visualized in Figure 6 and Figure 7, and detailed quantitative data are presented in Table 4, Table 5 and Table 6.

#### 3.3.1. Diagnostic Performance Enhancement

As illustrated in the radar chart (Figure 6), the integration of PUBS improved diagnostic separability across several flow-derived parameters. The PUBS-refined profile (blue line) generally extended beyond the Cascade-only profile (orange) across several hemodynamic parameters. Most notably, for Downward Mean Flow, the AUC increased from 0.747 using the Cascade-only method to 0.792 using the Cascade + PUBS method. This value was also numerically higher than the AUC obtained from manual reference-derived measurements for the same parameter. This suggests that signal refinement was particularly effective in capturing subtle diastolic flow variations that may be obscured by boundary-related segmentation variability. As a result, the method improved diagnostic separability by reducing boundary-related variability and preserving functionally coherent flow information relative to anatomical-only segmentation.

#### 3.3.2. Quantitative Accuracy and Mitigation of Partial Volume Effects

The PUBS algorithm reduced measurement error relative to the manual ground truth across a wide range of hemodynamic indices. This improvement was especially clear in velocity-derived metrics, where precise boundary definition was critical. The relative error analysis (Figure 7) further quantified this precision using the absolute percentage deviation from the manual ground truth within the SIH cohort.

Integrating physiological signal characteristics improved the accuracy of hemodynamic quantification, especially for boundary-sensitive metrics. The greatest improvement was seen in peak velocity measurements. The baseline anatomical segmentation showed a systematic deviation from the manual ground truth. This was likely due to peripheral non-flow voxels caused by partial volume effects. The PUBS algorithm used the pulsatile CSF signal to remove these static or inconsistent voxels at the vessel wall. As a result, the refined velocity was closer to the manual reference. The overall average relative error also decreased from 7.4% to 5.6%.

In summary, the initial anatomical segmentation provided a solid foundation. The addition of PUBS further improved quantitative agreement and supported better diagnostic separability in CSF hemodynamic assessment. This improvement was especially clear in the correction of velocity estimation under complex flow conditions.

#### 3.3.3. Sensitivity Analysis of PUBS Thresholding

**Figure 8:**
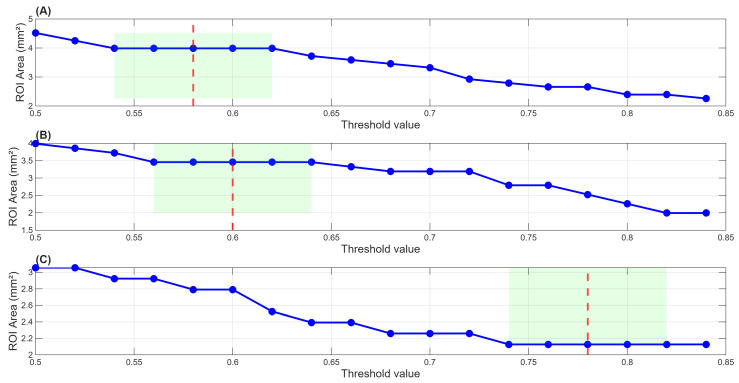
Sensitivity analysis of the PUBS threshold on aqueductal ROI area across representative clinical cohorts. The plots illustrate the relationship between the cross-correlation threshold value (x-axis) and the resulting ROI area in mm^2^ (y-axis). Panels (**A**–**C**) represent examples from the Normal controls, Pre-treatment SIH, and Post-treatment Recovery groups, respectively. A progressive reduction in ROI area is observed as the threshold increases, reflecting the exclusion of peripheral pixels that are more susceptible to partial-volume effects. The green shaded regions indicate the case-specific plateau regions automatically identified by the algorithm, within which the ROI area remains relatively stable against minor threshold perturbations. The red dashed lines indicate the optimal threshold $({Th}^{*}$) selected at the point of minimum calculated flow coefficient of variation (CV), supporting stable functional ROI definition and hemodynamic quantification.

To quantitatively evaluate the sensitivity of the PUBS thresholding step, we analyzed the relationship between the cross-correlation threshold values and the resulting aqueductal ROI areas across different clinical cohorts (see Figure 8). As illustrated, increasing the threshold naturally led to a progressive reduction in ROI area due to the mathematical exclusion of peripheral pixels. Crucially, a distinct “plateau region” was consistently observed across all cohorts. Within this optimal functional zone, the derived ROI area and subsequent flow measurements remained relatively stable against minor threshold perturbations (e.g., within a ±0.05 range). This visual evidence confirms the efficacy of the physiological refinement strategy detailed in Section 2.4, demonstrating that minimizing the flow CV successfully anchors the optimal threshold (${Th}^{*}$) in a highly stable region. Furthermore, this spatial stability may contribute to downstream waveform-extraction robustness. Because the core luminal ROI remains stable within the plateau, and because our feature extraction module strictly samples the top 5% peak velocity combined with Z-score normalization (as described in Section 2.7.2), the dynamic signal is expected to be less sensitive to minor boundary variations. Consequently, this sensitivity analysis supports the robustness of the PUBS-derived functional ROI against minor threshold fluctuations. Together with top 5% peak-velocity sampling and Z-score normalization, this design is expected to reduce the influence of boundary-level variations on downstream waveform feature extraction.

### 3.4. Deep Learning-Based Morphological Analysis

#### 3.4.1. Primary Validation of the End-to-End 1D-CNN Classifier

To evaluate whether waveform morphology alone could provide diagnostic discrimination between Normal controls and Pre-treatment SIH scans, the end-to-end 1D-CNN classifier was first assessed using repeated-seed participant-level grouped LOOCV. The ensemble prediction was generated by averaging the strictly out-of-sample SIH-class probabilities across 30 repeated-seed LOOCV runs for each participant.

**Figure 9:**
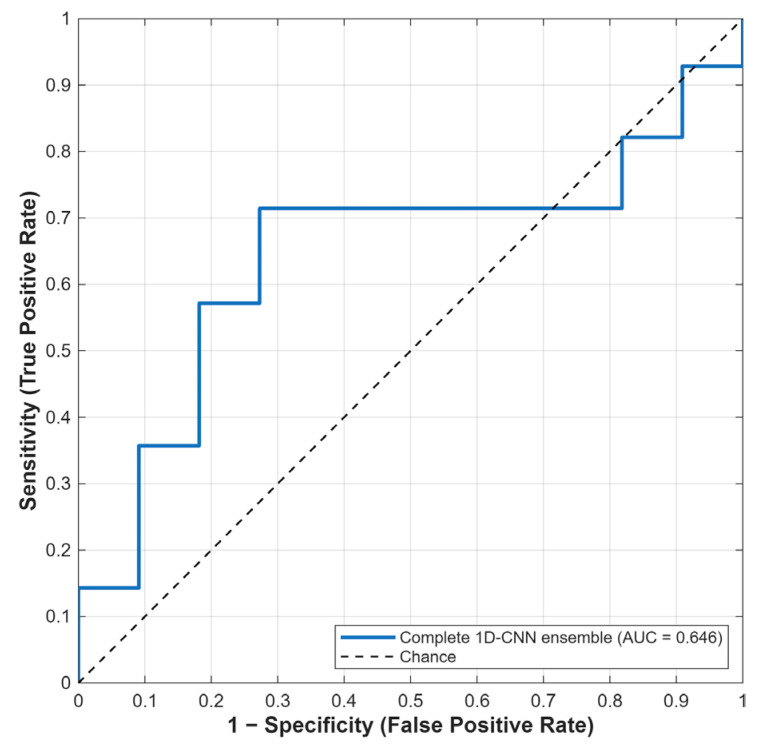
Primary validation ROC curve of the end-to-end 1D-CNN classifier. The ROC curve was generated from ensemble out-of-sample SIH probabilities averaged across 30 repeated-seed grouped LOOCV runs. The model showed modest discrimination between Normal controls and Pre-treatment SIH scans.

The repeated-seed ensemble ROC curve demonstrated modest out-of-sample discrimination, with an AUC of 0.646. The bootstrap 95% confidence interval was 0.455–0.826, and the permutation test showed a trend toward above-chance discrimination but did not reach conventional statistical significance (*p* = 0.072). At the optimal threshold determined by Youden’s Index, the classifier achieved a sensitivity of 71.4%, specificity of 72.7%, accuracy of 71.8%, and balanced accuracy of 72.1% (Table 7; Figure 9).

These findings indicate that the end-to-end 1D-CNN captured some SIH-related waveform information, but its classifier-level performance remained modest under strict out-of-sample validation. Therefore, the end-to-end 1D-CNN classifier was not interpreted as a validated stand-alone diagnostic model in the current cohort.

#### 3.4.2. Exploratory Latent-Space Visualization and Recovery Projection

After the primary LOOCV, a final baseline-trained 1D-CNN feature extractor was used to explore whether learned waveform representations showed group-level morphology patterns. This final model was trained using the complete baseline diagnostic cohort and was used only for exploratory latent feature extraction, PCA visualization, and Recovery projection. The learning curves of this final model showed stable convergence over 80 epochs (Supplementary Figure S4).

**Figure 10:**
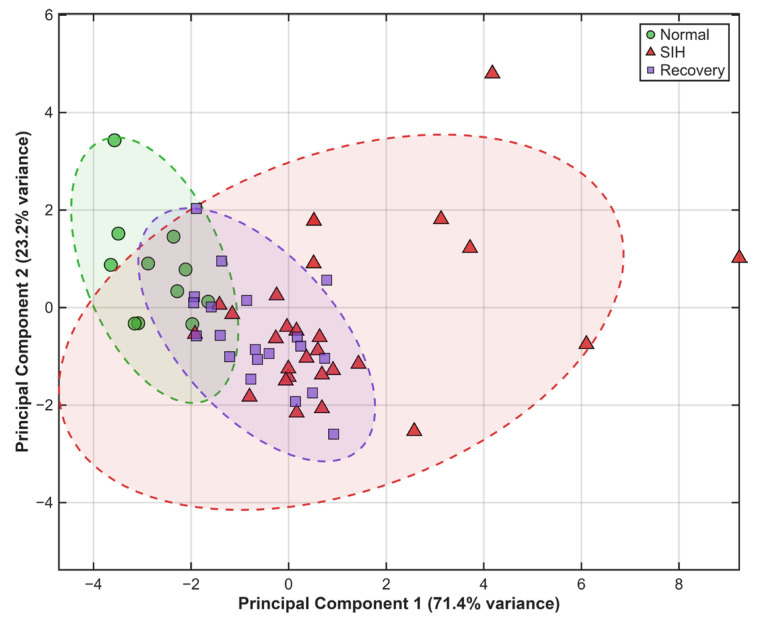
PCA projection of final baseline-trained 1D-CNN latent features. PCA was fitted using AI_Feat_1–AI_Feat_10 from the baseline diagnostic cohort, and Recovery scans were projected into the same PCA space. Normal controls and Pre-treatment SIH scans showed apparent separation mainly along PC1, while Recovery scans occupied an intermediate distribution.

PCA was performed on AI_Feat_1 to AI_Feat_10 using only the baseline diagnostic cohort. The first two principal components explained most of the latent-feature variance, with PC1 accounting for 71.4% and PC2 accounting for 23.2% of the total variance. Together, PC1 and PC2 explained 94.6% of the total variance. In the PCA space, Normal controls and Pre-treatment SIH scans showed apparent separation primarily along PC1, whereas Recovery scans occupied an intermediate distribution between the two baseline groups (Figure 10). PCA loadings showed that PC1 was mainly driven by positive contributions from AI_Feat_2, AI_Feat_6, AI_Feat_7, AI_Feat_8, and AI_Feat_9, and negative contributions from AI_Feat_3 and AI_Feat_4 (Supplementary Table S1).

This projection pattern suggests that the final baseline-trained latent space captured apparent waveform morphology differences between Normal and Pre-treatment SIH scans, and that Recovery scans tended to shift toward an intermediate or partially normalized morphology distribution. However, because this latent space was derived from a final model trained on the full baseline diagnostic cohort, these findings were interpreted as exploratory representation analysis rather than independent diagnostic validation.

#### 3.4.3. Distribution of AI-Derived Latent Features

**Figure 11:**
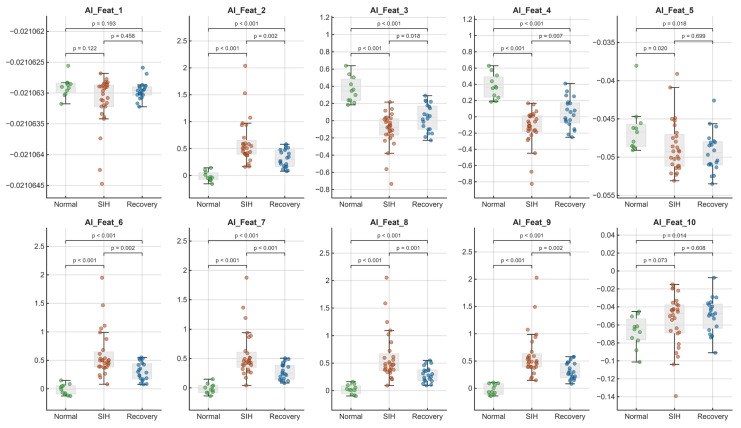
Distribution of exploratory 1D-CNN-derived latent features across clinical groups. Box-and-swarm plots show AI_Feat_1 to AI_Feat_10 extracted from the final baseline-trained 1D-CNN feature layer. Several latent features showed marked SIH-related shifts, with recovery scans generally showing intermediate distributions between Normal controls and Pre-treatment SIH scans. Pairwise *p*-values were calculated using the Mann–Whitney U test.

The distributions of AI_Feat_1 to AI_Feat_10 were further examined across Normal controls, Pre-treatment SIH, and Post-treatment Recovery groups using box-and-swarm plots (Figure 11). Several latent features showed marked group-wise differences. AI_Feat_2, AI_Feat_6, AI_Feat_7, AI_Feat_8, and AI_Feat_9 showed low values in Normal controls, increased values in Pre-treatment SIH scans, and intermediate values in Recovery scans. Pairwise comparisons showed significant differences between Normal and SIH for these features, as well as significant differences between SIH and Recovery, suggesting partial post-treatment shifts in the learned waveform representations.

In contrast, AI_Feat_3 and AI_Feat_4 showed the opposite directional pattern, with higher values in Normal controls, lower values in Pre-treatment SIH, and intermediate values in Recovery. AI_Feat_1 showed minimal variation across groups and did not demonstrate meaningful group separation. AI_Feat_5 and AI_Feat_10 showed relatively small absolute differences compared with the dominant latent features. Detailed group-wise values and pairwise *p*-values are summarized in Supplementary Table S2.

Overall, these feature-level patterns suggest that the final baseline-trained 1D-CNN captured exploratory waveform-shape representations that were associated with SIH status and recovery-related shifts. These findings were considered exploratory because the latent features were extracted from a final model trained on the complete baseline diagnostic cohort.

#### 3.4.4. Exploratory Apparent ROC Analysis

**Figure 12:**
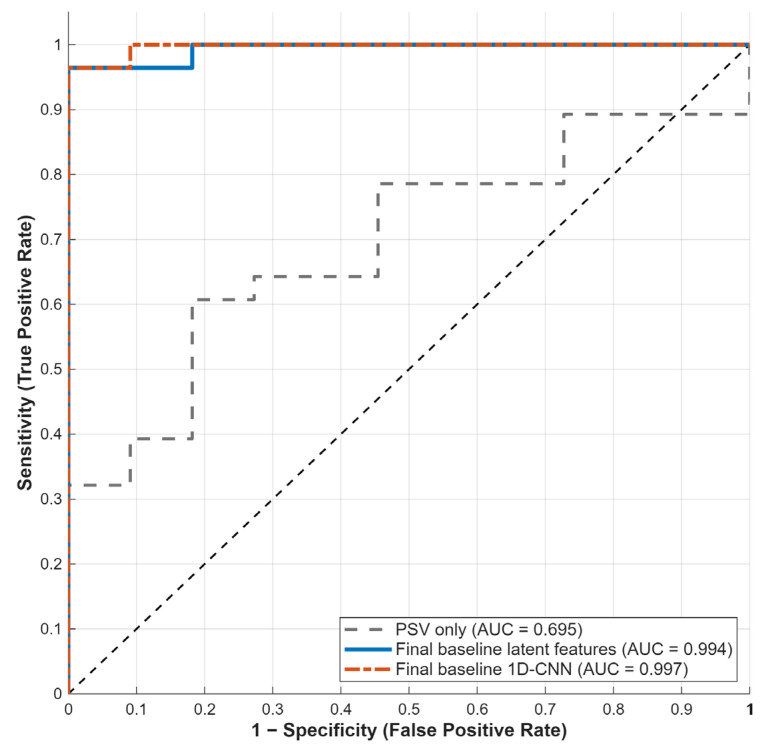
Exploratory apparent ROC analysis of PSV and final baseline-trained 1D-CNN-derived features. ROC curves compare PSV, final baseline-trained latent features, and final baseline-trained 1D-CNN probability for Normal-versus-SIH separability. These analyses represent apparent separability from the final baseline-trained model and were not interpreted as independent validation.

As a secondary exploratory analysis, the apparent Normal-versus-SIH separability of conventional PSV, final baseline-trained latent features, and the final baseline-trained 1D-CNN probability was evaluated. PSV alone achieved an AUC of 0.695. In comparison, the final baseline-trained latent-feature model achieved an apparent AUC of 0.994, and the final baseline-trained 1D-CNN softmax probability achieved an apparent AUC of 0.997 (Supplementary Table S3; Figure 12).

These exploratory ROC results indicate that the final baseline-trained latent space showed strong apparent separation between Normal and Pre-treatment SIH scans. However, because the latent features and final-model probabilities were derived from a model trained on the complete baseline diagnostic cohort, these AUC values were not interpreted as independent out-of-sample diagnostic performance. The primary classifier-level validation remained the repeated-seed grouped LOOCV result described above.

## 4. Discussion

### 4.1. Principal Findings and Overall Contributions

This study presents a fully automated, end-to-end framework for cine PC-MRI analysis of cerebral aqueduct CSF hemodynamics in spontaneous intracranial hypotension (SIH). The proposed pipeline integrates (1) a cascade localization strategy using YOLOv4 for robust midbrain/aqueduct targeting, (2) aqueduct segmentation using a multi-scale CNN (MultiResUNet), (3) physiology-informed refinement via pulsatility-based segmentation (PUBS), and (4) morphology-driven waveform feature learning using a 1D-CNN trained on 32-point cardiac-cycle velocity waveforms, with repeated-seed participant-level grouped LOOCV used for primary classifier validation. This workflow addresses an important clinical need for objective and reproducible tools to support SIH diagnosis and treatment monitoring, beyond conventional qualitative MRI signs and subjective symptom reporting [4,5]. Moreover, it operationalizes the concept that SIH is not merely CSF volume depletion but also a disorder of altered craniospinal biomechanics and compliance that can manifest in dynamic CSF flow signatures [6,7,8,9].

Three contributions emerge from the Results. First, the cascade localization–segmentation design substantially improves segmentation robustness compared with a full-image baseline, particularly under extreme class imbalance and low contrast typical of the aqueduct in SIH. Across cohorts, the cascade design improves both accuracy and robustness, yielding higher DSC and markedly lower HD95. The robustness gain is most evident in Pre-treatment SIH, where the full-image model shows extreme boundary outliers (HD95 of 15.37 ± 44.98 px), while the cascade model remains stable (HD95 of 1.66 ± 0.74 px) (Table 2, Figure 5). Second, PUBS refinement improves the fidelity of flow quantification by suppressing boundary noise and partial volume–driven errors. In the SIH cohort, the mean relative error decreases from 7.4% to 5.6%, and the radar plot shows broader AUC profiles across several hemodynamic parameters. Notably, the AUC for downward mean flow improves from 0.747 to 0.792, numerically higher than the manual-reference AUC of 0.750 (Figure 6 and Figure 7). Third, the waveform morphology analysis provides exploratory evidence that SIH-related information may be encoded in the temporal shape of aqueductal CSF velocity waveforms. Under repeated-seed participant-level grouped LOOCV, the end-to-end 1D-CNN classifier shows modest out-of-sample discrimination rather than robust stand-alone diagnostic performance. However, exploratory analysis of the final baseline-trained latent feature space demonstrates marked, apparent Normal-versus-SIH separability and an intermediate recovery distribution in PCA space. This discrepancy suggests that waveform morphology may contain physiologically meaningful SIH-related information, but larger independent cohorts are required before 1D-CNN-derived features can be considered validated diagnostic or monitoring biomarkers.

### 4.2. Challenges of Aqueduct Segmentation in SIH Cine PC-MRI

Aqueduct segmentation in PC-MRI is inherently challenging because the aqueduct occupies only a minute fraction of the image and is susceptible to partial volume effects and ROI-definition variability [12,13,14,17]. These difficulties are further amplified in SIH, where aqueductal CSF velocities and flow measures are reduced [13]. As a result, unlike hyperdynamic conditions (e.g., hydrocephalus or NPH/iNPH), SIH often provides limited flow-related contrast. These characteristics help explain the failure patterns observed in the full-image baseline: the model must simultaneously identify the target location and delineate its boundary under extreme class imbalance, increasing the risk of off-target false positives that inflate boundary-based errors (e.g., HD95) and compromise downstream quantification (Table 2, Figure 5).

Previous investigations into aqueductal CSF dynamics, such as those by Capel et al. [9] and Spijkerman et al. [16], have typically relied on manual or semi-automated ROI definition prior to detailed segmentation to ensure measurement reliability. This dependence on manual localization underscores a critical insight: accurately delineating the aqueduct from the complex midbrain background requires constraining the search space to exclude physiological artifacts. Our cascade design automates this essential ‘spatial attention’ mechanism by implementing YOLOv4 [20,21] as a preliminary localizer. By explicitly constraining the ROI, this detection stage effectively filters out background noise and decouples localization from segmentation. Consequently, this allowed the subsequent MultiResUNet [22] to focus on delineating the micro-tubular aqueduct within a constrained anatomical region, thereby approximating the stabilizing effect of manual ROI placement. As demonstrated in Table 2, this methodological advantage translates into statistically significant improvements in segmentation performance. The proposed cascade model significantly outperforms the full-image model across all metrics (p < 0.05).

### 4.3. The Role of PUBS: From Anatomical Masks to Functional ROIs

PC-MRI quantification of aqueductal CSF flow is highly sensitive to ROI definition. Peak-related metrics and local extrema are particularly vulnerable to boundary placement and partial volume effects, which can introduce systematic measurement bias [15,17,31]. Consistent with this, our ablation analysis indicates that an anatomically plausible mask alone (cascade-only segmentation) does not guarantee reliable velocity estimation, whereas PUBS refinement reduces boundary-related bias and improves agreement with the manual reference, reducing the mean relative error in the SIH cohort from 7.4% to 5.6% (≈24% relative reduction). This finding also echoes prior semiautomatic work showing that anatomically reasonable ROIs may still yield physiologically unreliable aqueduct flow measurements when boundary uncertainty is present [17].

PUBS addresses this limitation by exploiting temporal consistency across the cardiac cycle to identify functional flow pixels, rather than relying solely on anatomical intensity information [18]. In our pipeline, PUBS serves as a physiology-informed post-processing layer after deep segmentation, suppressing boundary contamination and improving measurement fidelity without manual threshold tuning. The benefit is evident in both reduced quantification error (Figure 7) and improved diagnostic separability across hemodynamic parameters, reflected by a broader radar-chart profile after PUBS refinement across several parameters (Figure 6). Together, these results support PUBS as a complementary component that improves both quantitative agreement and diagnostic separability.

Furthermore, the automated identification of the PUBS plateau may reduce the influence of boundary-level segmentation variation on downstream waveform extraction. Combined with top 5% peak-velocity sampling and Z-score normalization, this design was intended to improve the robustness of morphology-based feature extraction by emphasizing temporally coherent luminal flow signals rather than boundary-dependent peripheral pixels.

### 4.4. Background Correction and Consistency of Hemodynamic Parameters with Prior PC-MRI Studies

Accurate quantification of aqueductal CSF hemodynamics in PC-MRI depends not only on robust ROI delineation but also on appropriate correction of background phase offsets. Phase errors caused by system imperfections and eddy currents can introduce systematic bias in velocity and flow estimates, particularly for small structures such as the cerebral aqueduct; therefore, background phase correction is widely regarded as an essential step for reliable quantification [29,30]. In this work, we apply a fully automated local background correction using static tissue adjacent to the aqueduct, as described in Section 2.5 and illustrated in Figure 3. The midbrain reference region is divided into an 8 × 8 grid, and cells overlapping a dilated aqueduct exclusion zone (disk radius = 6 pixels) are discarded. We then select the eight cells with the lowest temporal SD (t-SD) across 32 cardiac phases as the static reference and subtract their per-frame mean phase velocity from the aqueduct waveform to correct spatially varying phase offsets.

With background correction in place, the hemodynamic parameters derived from our automated pipeline show patterns consistent with prior aqueduct PC-MRI studies in SIH. Notably, Tung et al. reported reduced aqueductal peak velocity and flow-related measures in SIH compared with controls, with improvement after recovery [13], and similar SIH-related alterations have been described in other PC-MRI reports [6]. In agreement, we observe significant group differences between SIH and controls across multiple velocity- and flow-based parameters, including Downward peak velocity, Upward/Downward CSF total flow, Absolute stroke volume, and peak/mean flow measures (Table 6), with most parameters trending toward normalization in the recovery cohort. This concordance supports the validity of our parameter estimation and indicates that the proposed workflow preserves clinically meaningful physiological information while eliminating manual processing steps. Beyond consistency with prior SIH findings, our results directly address reproducibility concerns arising from software-dependent differences in aqueduct flow quantification [32], supporting more standardized and comparable measurements across analyses.

By integrating local background correction with physiology-informed ROI refinement and fully automated hemodynamic parameter estimation, our workflow improves measurement stability and supports reproducible longitudinal assessment.

### 4.5. Beyond Scalar Metrics: Exploratory Waveform Morphology Analysis Using 1D-CNN

Most aqueductal PC-MRI studies summarize CSF dynamics using scalar parameters, such as peak velocity, mean flow, or stroke volume [12,13,14,15]. Although these metrics are clinically interpretable and physiologically meaningful, they compress the cardiac-cycle waveform into a limited set of values and may overlook temporal morphology information. In this study, we therefore explore whether the shape of the 32-point aqueductal CSF velocity waveform contains additional SIH-related information beyond conventional scalar measurements.

Under the strict repeated-seed participant-level grouped LOOCV framework, the end-to-end 1D-CNN classifier demonstrated only modest out-of-sample diagnostic performance. The ensemble AUC was 0.646, with a bootstrap 95% confidence interval of 0.455–0.826 and a permutation-test *p*-value of 0.072 (Figure 9, Table 7). Although sensitivity and specificity were balanced at approximately 70%, the wide confidence interval and non-significant permutation test indicate that the classifier-level generalizability of the current 1D-CNN remains limited. Therefore, the end-to-end 1D-CNN should not be interpreted as a validated stand-alone diagnostic model at this stage.

Nevertheless, the final baseline-trained latent-space analysis suggests that waveform morphology may still encode SIH-related physiological information. PCA of the final baseline-trained latent features shows that the first two principal components explain most of the latent-feature variance, with apparent separation between Normal controls and Pre-treatment SIH scans mainly along PC1. Post-treatment Recovery scans tend to occupy an intermediate distribution between the two baseline groups (Figure 10). This pattern suggests that Post-treatment Recovery scans may not simply revert to the Normal cluster but instead may represent a transitional waveform morphology state, potentially reflecting partial restoration of intracranial CSF dynamics after treatment.

The distribution of individual AI-derived latent features further supports this interpretation. Several features, including AI_Feat_2, AI_Feat_6, AI_Feat_7, AI_Feat_8, and AI_Feat_9, show low values in Normal controls, higher values in Pre-treatment SIH scans, and intermediate values in Recovery scans. Conversely, AI_Feat_3 and AI_Feat_4 show the opposite directional pattern (Figure 11). These bidirectional feature patterns suggest that the 1D-CNN does not simply capture a single amplitude-like descriptor but instead learns a multi-dimensional representation of waveform morphology. However, because these features are extracted from a final model trained on the complete baseline diagnostic cohort, they should be interpreted as exploratory latent-space findings rather than independently validated diagnostic biomarkers.

The exploratory ROC analysis also highlights this distinction. PSV alone shows moderate apparent separability, whereas the final baseline-trained latent features and final 1D-CNN probability show markedly higher apparent AUC values (Figure 12). However, these high AUCs reflect apparent separation within the final baseline-trained representation space and should not be compared directly with the repeated-seed grouped LOOCV result as independent validation. Instead, the discrepancy between modest out-of-sample classifier performance and strong final latent-space separability suggests that SIH-related waveform morphology may be present but remains difficult to generalize reliably in a small and imbalanced cohort.

1D-CNNs are well suited for hierarchical representation learning in biomedical time-series analysis and have demonstrated strong performance in ECG waveform classification, where subtle shape differences are clinically meaningful [25,26,27]. Taken together, these findings support a cautious interpretation: Aqueductal CSF waveform morphology may contain physiologically relevant information beyond scalar flow metrics, but the current 1D-CNN-derived features should be considered hypothesis-generating biomarkers [33]. Larger independent cohorts are required to determine whether these latent waveform patterns are reproducible, physiologically interpretable, and clinically useful for SIH diagnosis or post-treatment monitoring.

### 4.6. Limitations and Future Work

Several limitations merit discussion. First, assembling a large SIH dataset is practically constrained by both the disease’s relative rarity and the high natural attrition rate in clinical follow-up. In routine clinical practice, successfully treated patients (e.g., those who experience significant symptom relief following an Epidural Blood Patch) often do not return for follow-up MRI scans. As a result, this was a single-center retrospective study with a modest sample size (controls: 11; SIH: 28; recovery: 20) and a fixed acquisition protocol. To reduce the risk of seed-selection bias in the small-sample 1D-CNN analysis, we used repeated-seed participant-level grouped LOOCV and reported the ensemble out-of-sample performance rather than selecting a best-performing seed. However, the resulting classifier performance remains modest, emphasizing the need for larger validation cohorts. In contrast to this small-sample limitation of the 1D-CNN analysis, the risk of clinical-cohort data leakage in the upstream anatomical components was minimized by training the Tiny YOLOv4 and MultiResUNet models on completely separate, independent 100-subject datasets with no overlap with the clinical analysis cohort.

Second, there was a statistically significant age difference between SIH patients and controls (*p* = 0.036). While previous studies in healthy cohorts [14] suggest that baseline aqueductal CSF flow is relatively stable across ages, aging is known to alter tissue elasticity and compliance. Therefore, we cannot fully exclude age as a potential confounder in the biomechanical differences observed between the SIH and control groups. Although the longitudinal Post-treatment Recovery subset reduces this concern for treatment-related comparisons because recovery scans were obtained from the same SIH patients, the observed shifts in exploratory latent features should still be interpreted cautiously. Larger age-matched validation studies are needed to confirm the relationship between these waveform morphology changes, SIH pathophysiology, and treatment response. To address these limitations, future work should proceed in a staged manner: first, by expanding the sample size within our institution to reinforce statistical power and evaluate the stability of the candidate waveform morphology features; and subsequently, by conducting multi-center data collection to rigorously assess the model’s generalizability across different scanners and populations.

Third, all imaging was acquired on a single platform with a single protocol (1.5T, 5 mm slice thickness, venc 20 cm/s). Given the extremely small aqueduct size, equipment-dependent noise can significantly alter the extracted waveform morphology. While 1.5T systems typically yield fewer susceptibility artifacts at the skull base compared to 3.0T, future work should rigorously evaluate the model’s generalizability. Specifically, it is necessary to investigate whether the learned waveform features remain robust under different venc settings, across equipment from different manufacturers, and in recent 3.0T MRI systems, as field-strength–dependent differences have been well-documented in other CSF-related contexts [34].

Fourth, manual delineation was primarily established by an experienced neuroradiologist as the reference standard. While manual profiling of tiny structures like the cerebral aqueduct is inherently susceptible to observer subjectivity [15,17,31], our comprehensive inter-rater analysis involving a second independent expert reader across the entire dataset demonstrated near-perfect consistency (average DSC > 0.98 and HD95 < 0.1 mm in all groups). This high agreement reduced the likelihood of major observer-dependent bias and supported the reliability of the reference masks used for downstream analysis.

Finally, the 1D-CNN waveform morphology analysis remains exploratory. Although repeated-seed grouped LOOCV was used to reduce seed-selection bias, the end-to-end 1D-CNN classifier shows only modest out-of-sample discrimination and did not reach conventional statistical significance in permutation testing. In addition, the final baseline-trained latent-feature analyses represent apparent separability rather than independent validation because the feature extractor was trained on the complete baseline diagnostic cohort. Larger independent cohorts are required to confirm the reproducibility and physiological meaning of these waveform morphology features. Comprehensive standardized symptom scores, quantitative imaging indices, and long-term treatment-response data were not systematically available in the present retrospective dataset. Future studies should correlate the exploratory waveform morphology features with clinical and physiological measures, including lumbar puncture opening pressure, quantitative brain sagging indices, HIT-6 headache scores, recurrence rates, and treatment response [2,3,4,5]. Such validation is necessary before these features can be used for prognostic modeling or prediction of epidural blood patching response.

## 5. Conclusions

This study presents a fully automated end-to-end framework for analyzing aqueductal CSF hemodynamics in spontaneous intracranial hypotension (SIH). By combining cascade localization–segmentation with physiology-informed pulsatility-based refinement (PUBS), the pipeline reduces partial volume effects and enables accurate flow quantification even under low-contrast conditions. Exploratory 1D-CNN-based waveform morphology analysis further suggests that SIH-related information may be encoded in temporal waveform shape beyond conventional scalar flow metrics. However, the modest out-of-sample performance of the end-to-end 1D-CNN classifier indicates that these morphology-derived features should currently be interpreted as exploratory biomarker candidates rather than validated stand-alone diagnostic tools. Larger independent cohorts are required to confirm their reproducibility, physiological meaning, and potential utility for SIH diagnosis, longitudinal monitoring, and treatment-response prediction.

## Figures and Tables

**Table 1:** Demographic characteristics of the study population.

Characteristic	Normal Controls	Pre-Treatment SIH	Post-Treatment Recovery	*p*-Value
Number of examinations	11	28	20	
Age (years)	32.9 ± 5.3	40.1 ± 10.4	40.1 ± 11.1	0.036 †
Sex (Male/Female)	6/5	7/21	4/16	0.13 ‡

The Post-treatment Recovery group was a subset of the Pre-treatment SIH group. Data are presented as Mean ± SD or Count. † *p*-value was calculated for Normal controls versus Pre-treatment SIH using Student’s *t*-test. ‡ *p*-value was calculated for Normal controls versus Pre-treatment SIH using Fisher’s exact test.

**Table 2:** Segmentation performance.

Population Group	Metric	Cascade Model	Full-Image Model	*p*-Value
Normal controls	Dice (DSC) HD95 (px)	0.82 ± 0.07 1.39 ± 0.34	0.69 ± 0.10 1.98 ± 0.56	**0.0098** **0.0186**
Pre-treatment SIH	Dice (DSC) HD95 (px)	0.57 ± 0.35 1.66 ± 0.74	0.45 ± 0.29 15.37 ± 44.98	**0.0230** **0.0019**
Post-treatment Recovery	Dice (DSC) HD95 (px)	0.79 ± 0.18 1.23 ± 0.31	0.61 ± 0.17 2.01 ± 0.30	**<0.001** **<0.001**

Data are presented as mean ± SD. *p*-values were calculated using the Wilcoxon signed-rank test for paired comparison between the cascade model and the full-image model within each clinical group, with statistically significant values (*p* < 0.05) highlighted in bold. HD95 values are reported in pixels because the ablation analysis was designed to compare model performance within the same image-coordinate space. The in-plane pixel size was approximately 0.365 mm/pixel based on the acquisition matrix and field of view.

**Table 3:** Inter-rater reliability metrics between two independent expert annotators across clinical cohorts.

Population Group	DSC	IoU	HD95 (mm)
Normal controls	0.9883 ± 0.0235	0.9778 ± 0.0444	0.0829 ± 0.1495
Pre-treatment SIH	0.9899 ± 0.0254	0.9812 ± 0.0469	0.0601 ± 0.1318
Post-treatment Recovery	0.9965 ± 0.0133	0.9934 ± 0.0251	0.0255 ± 0.0862

For inter-rater reliability analysis, HD95 values are reported in millimeters to reflect the physical boundary discrepancy between expert manual annotations and to facilitate clinical interpretation.

**Table 4:** Quantitative hemodynamic parameters derived from expert manual segmentation.

Parameter	Normal (*n* = 11)	SIH (*n* = 28)	Recovery (*n* = 20)	*p* (Norm vs. SIH)	AUC (Norm vs. SIH)	*p* (Norm vs. Recovery)
Upward Mean Flow (mL/s)	0.063 ± 0.031	0.042 ± 0.050	0.062 ± 0.035	**0.013**	0.760	1.000
Downward Mean Flow (mL/s)	−0.068 ± 0.032	−0.048 ± 0.061	−0.067 ± 0.035	**0.017**	0.750	0.984
Total Mean Flow (mL/s)	0.131 ± 0.062	0.089 ± 0.110	0.129 ± 0.070	**0.011**	0.770	0.853
Upward Peak Velocity (cm/s)	6.579 ± 1.722	5.465 ± 3.007	6.894 ± 2.627	0.089	0.679	0.665
Downward Peak Velocity (cm/s)	−7.726 ± 2.000	−5.940 ± 3.306	−7.916 ± 2.316	**0.041**	0.714	0.757
Upward Peak Flow (mL/s)	0.098 ± 0.049	0.068 ± 0.073	0.095 ± 0.051	**0.017**	0.750	0.951
Downward Peak Flow (mL/s)	−0.115 ± 0.050	−0.079 ± 0.093	−0.114 ± 0.061	**0.014**	0.756	0.820
Summation of Peak Flow (mL/s)	0.213 ± 0.098	0.147 ± 0.165	0.209 ± 0.109	**0.017**	0.750	0.918
Upward CSF total flow (mL/cycle)	1.012 ± 0.498	0.696 ± 0.897	1.000 ± 0.557	**0.013**	0.760	0.918
Downward CSF total flow (mL/cycle)	−1.072 ± 0.497	−0.728 ± 0.843	−1.053 ± 0.569	**0.014**	0.756	0.885
Absolute stroke volume (mL/cycle)	2.084 ± 0.989	1.425 ± 1.729	2.052 ± 1.117	**0.010**	0.769	0.918

Data are presented as Mean ± SD. Statistically significant values (*p* < 0.05) were highlighted in bold. (Mann–Whitney U test). AUC (Area Under the Curve).

**Table 5:** Quantitative hemodynamic parameters derived from baseline automated anatomical segmentation.

Parameter	Normal (*n* = 11)	SIH (*n* = 28)	Recovery (*n* = 20)	*p* (Norm vs. SIH)	AUC (Norm vs. SIH)	*p* (Norm vs. Recovery)
Upward Mean Flow (mL/s)	0.066 ± 0.029	0.045 ± 0.052	0.063 ± 0.035	**0.012**	0.763	0.757
Downward Mean Flow (mL/s)	−0.070 ± 0.033	−0.050 ± 0.060	−0.068 ± 0.035	**0.018**	0.747	1.000
Total Mean Flow (mL/s)	0.136 ± 0.061	0.096 ± 0.112	0.132 ± 0.068	**0.009**	0.773	0.918
Upward Peak Velocity (cm/s)	6.579 ± 1.722	5.816 ± 3.079	6.932 ± 2.582	0.175	0.643	0.665
Downward Peak Velocity (cm/s)	−7.726 ± 2.000	−6.168 ± 3.169	−7.918 ± 2.312	0.063	0.695	0.757
Upward Peak Flow (mL/s)	0.109 ± 0.045	0.075 ± 0.073	0.099 ± 0.051	**0.005**	0.792	0.757
Downward Peak Flow (mL/s)	−0.120 ± 0.049	−0.086 ± 0.092	−0.116 ± 0.060	**0.020**	0.744	0.665
Summation of Peak Flow (mL/s)	0.229 ± 0.093	0.161 ± 0.164	0.214 ± 0.108	**0.008**	0.779	0.635
Upward CSF total flow (mL/cycle)	1.059 ± 0.480	0.754 ± 0.949	1.029 ± 0.551	**0.010**	0.769	0.984
Downward CSF total flow (mL/cycle)	−1.119 ± 0.512	−0.788 ± 0.839	−1.064 ± 0.556	**0.018**	0.747	0.984
Absolute stroke volume (mL/cycle)	2.178 ± 0.971	1.542 ± 1.763	2.093 ± 1.093	**0.011**	0.766	0.853

Data are presented as Mean ± SD. Statistically significant values (*p* < 0.05) were highlighted in bold. (Mann–Whitney U test). AUC (Area Under the Curve).

**Table 6:** Quantitative hemodynamic parameters derived from proposed PUBS-refined segmentation.

Parameter	Normal (*n* = 11)	SIH (*n* = 28)	Recovery (*n* = 20)	*p* (Norm vs. SIH)	AUC (Norm vs. SIH)	*p* (Norm vs. Recovery)
Upward Mean Flow (mL/s)	0.067 ± 0.031	0.045 ± 0.050	0.063 ± 0.034	**0.008**	0.776	0.757
Downward Mean Flow (mL/s)	−0.076 ± 0.032	−0.051 ± 0.062	−0.071 ± 0.037	**0.005**	0.792	0.635
Total Mean Flow (mL/s)	0.143 ± 0.062	0.095 ± 0.111	0.134 ± 0.070	**0.008**	0.779	0.635
Upward Peak Velocity (cm/s)	6.610 ± 1.671	5.552 ± 2.996	6.919 ± 2.604	0.115	0.666	0.695
Downward Peak Velocity (cm/s)	−7.758 ± 1.955	−5.988 ± 3.274	−7.918 ± 2.312	**0.041**	0.714	0.757
Upward Peak Flow (mL/s)	0.105 ± 0.048	0.073 ± 0.074	0.099 ± 0.051	**0.016**	0.753	0.820
Downward Peak Flow (mL/s)	−0.129 ± 0.050	−0.085 ± 0.096	−0.121 ± 0.062	**0.005**	0.792	0.635
Summation of Peak Flow (mL/s)	0.234 ± 0.097	0.157 ± 0.169	0.220 ± 0.110	**0.008**	0.779	0.695
Upward CSF total flow (mL/cycle)	1.081 ± 0.505	0.739 ± 0.898	1.031 ± 0.562	**0.010**	0.769	0.726
Downward CSF total flow (mL/cycle)	−1.207 ± 0.497	−0.782 ± 0.872	−1.103 ± 0.561	**0.009**	0.773	0.665
Absolute stroke volume (mL/cycle)	2.288 ± 0.993	1.521 ± 1.753	2.134 ± 1.112	**0.008**	0.779	0.665

Data are presented as Mean ± SD. Statistically significant values (*p* < 0.05) were highlighted in bold. (Mann–Whitney U test). AUC (Area Under the Curve).

**Table 7:** Primary validation of the end-to-end 1D-CNN classifier using repeated-seed grouped LOOCV.

Model	Validation Status	AUC	95% CI	*p*-Value	Sensitivity	Specificity	Accuracy	Balanced Accuracy
End-to-end 1D-CNN ensemble	Repeated-seed grouped LOOCV	0.646	0.455–0.826	0.072	71.4	72.7	71.8	72.1

Ensemble performance was calculated by averaging out-of-sample SIH probabilities across 30 repeated-seed grouped LOOCV runs. The AUC confidence interval was estimated by bootstrap resampling, and the *p*-value was obtained by permutation testing. No best-performing seed was selected. Sensitivity, Specificity, Accuracy, and Balanced accuracy are reported as percentages.

## Data Availability

The data presented in this study are available from the corresponding author upon reasonable request. Data sharing is subject to Institutional Review Board restrictions and privacy regulations.

## Supplementary Materials

The following supporting information can be downloaded at: https://www.mdpi.com/article/10.3390/diagnostics16121939/s1, Figure S1: Training and validation loss curves of the Tiny YOLOv4 midbrain localization model; Figure S2: Precision–recall curve of the Tiny YOLOv4 midbrain localization model on the independent test set; Figure S3: Training and validation curves of the MultiResUNet cascade segmentation model; Figure S4: Learning curves of the final baseline-trained 1D-CNN feature extractor; Table S1: PCA loadings of AI_Feat_1 to AI_Feat_10 for the final baseline-trained latent-feature space; Table S2: Group-wise distributions and pairwise comparisons of AI_Feat_1 to AI_Feat_10 among Normal controls, Pre-treatment SIH, and Post-treatment Recovery scans; Table S3: Exploratory apparent ROC analysis of PSV, final baseline-trained latent-feature model, and final baseline-trained 1D-CNN softmax probability for Normal-versus-SIH separability.
