# Supplementary material for: Automated Aqueductal CSF Flow Analysis in Spontaneous Intracranial Hypotension: Hemodynamic Quantification and Exploratory Waveform Morphology Assessment Using Cine PC-MRI
Source: Diagnostics (Basel). 2026 Jun 22;16(12):1939. doi: 10.3390/diagnostics16121939 (PMC13297683; doi:10.3390/diagnostics16121939)
Supplement: Supplementary file 1 [file diagnostics-16-01939-s001.zip › diagnostics-4230881-supplementary.pdf]

Supplementary Materials: Automated Aqueductal CSF Flow Analysis in Spontaneous Intracranial Hypotension: Hemodynamic Quantification and Exploratory Waveform Morphology Assessment Using Cine PC-MRI

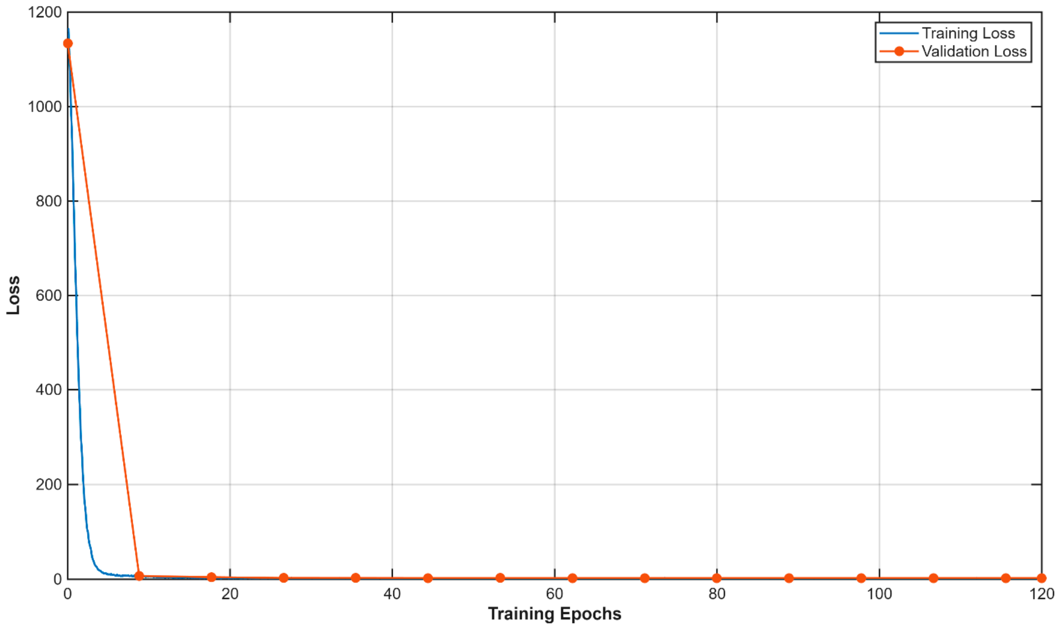

Figure S1. Learning curves of the Tiny YOLOv4 localizer. The training and validation loss are plotted as a function of training epochs (up to 120 epochs). The curves demonstrate rapid model convergence within the initial epoch and maintain stability without evidence of overfitting.

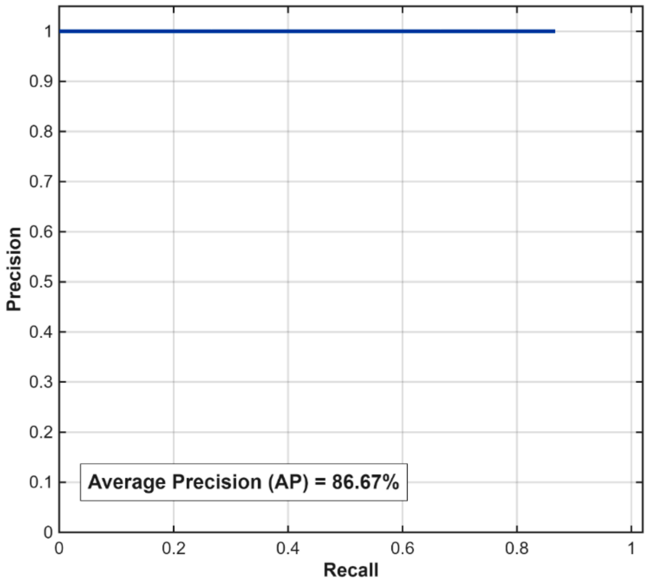

Figure S2. Precision-Recall (PR) curve of the Tiny YOLOv4 localizer evaluated on the independent test set. The model achieved an Average Precision (AP) of 86.67%. Notably, the localizer maintained a perfect precision of 1.000 across all operating points up to a recall of 0.867, indicating zero false-positive detections in the test cohort.

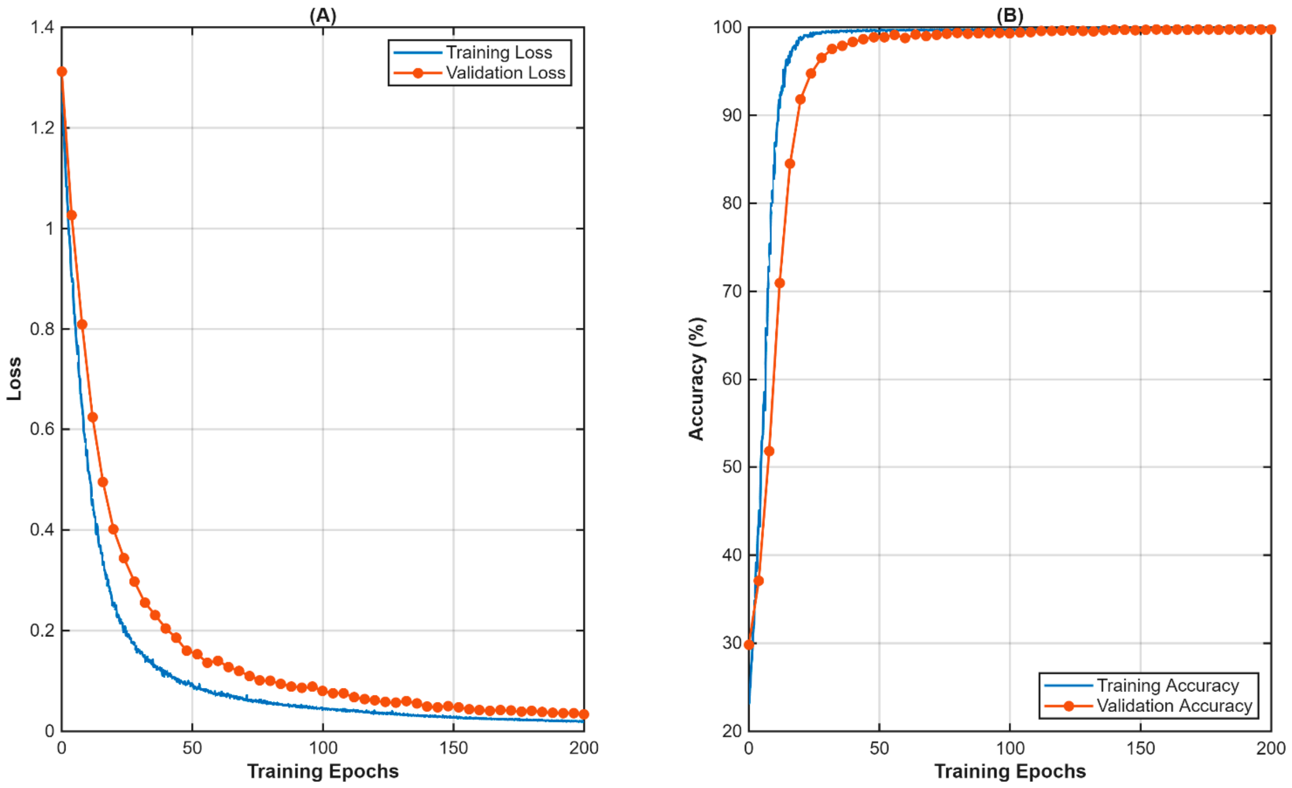

Figure S3. Learning curves of the MultiResUNet segmentation network. The subplots illustrate the training and validation loss (A), alongside the segmentation accuracy (B), over a 200-epoch training period. The curves demonstrate rapid and stable model convergence, with accuracy steeply climbing and plateauing at nearly 100% after approximately 50 epochs. The validation curves closely tracked the training curves throughout the training process, suggesting stable learning behavior without obvious overfitting.

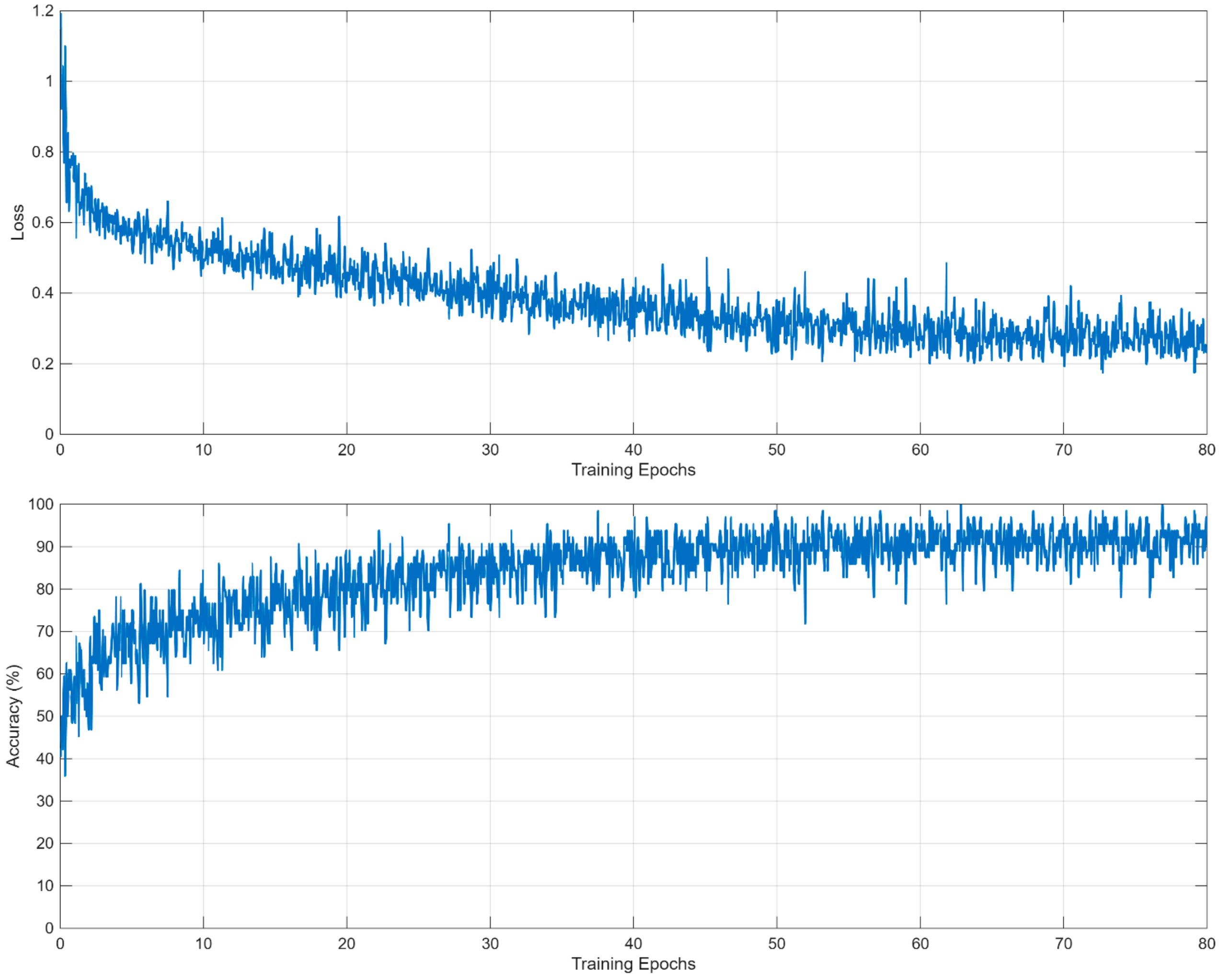

Figure S4. Learning curves of the final baseline-trained 1D-CNN feature extractor. Training loss and training accuracy are shown over 80 epochs. The loss progressively decreased, while the training accuracy increased and stabilized, indicating stable convergence of the final baseline-trained 1D-CNN model. This model was subsequently used for exploratory latent feature extraction, PCA projection, and recovery projection.

Supplementary Table S1. PCA loadings of exploratory 1D-CNN-derived latent features.

Feature	PC1 loading	PC2 loading
AI_Feat_1	-0.26477473	-0.33561954
AI_Feat_2	0.370814173	-0.028056648
AI_Feat_3	-0.342111395	0.228083745
AI_Feat_4	-0.350872791	0.206928432
AI_Feat_5	-0.005850851	0.628885472
AI_Feat_6	0.37253152	0.019287219
AI_Feat_7	0.369793104	0.039973973
AI_Feat_8	0.369085989	0.079328748
AI_Feat_9	0.372519857	-0.002530413
AI_Feat_10	-0.041022584	-0.622867942

PCA was fitted using the baseline diagnostic cohort only. Recovery scans were projected into the same PCA space without influencing the PCA axes. Loadings indicate the contribution of each AI-derived latent feature to PC1 and PC2.

Supplementary Table S2. Group-wise distributions and pairwise comparisons of exploratory 1D-CNN-derived latent features.

Feature	Normal	SIH	Recovery	Normal vs SIH p-value	Normal vs Recovery p-value	SIH vs Recovery p-value
AI_Feat_1	-0.021 ± 0.000	-0.021 ± 0.000	-0.021 ± 0.000	0.122	0.193	0.458
AI_Feat_2	-0.015 ± 0.089	0.616 ± 0.408	0.318 ± 0.166	<0.001	<0.001	0.002
AI_Feat_3	0.362 ± 0.149	-0.104 ± 0.201	0.035 ± 0.156	<0.001	<0.001	0.018
AI_Feat_4	0.374 ± 0.151	-0.136 ± 0.220	0.048 ± 0.183	<0.001	<0.001	0.007
AI_Feat_5	-0.046 ± 0.003	-0.049 ± 0.003	-0.049 ± 0.003	0.020	0.018	0.699
AI_Feat_6	-0.012 ± 0.091	0.600 ± 0.401	0.304 ± 0.160	<0.001	<0.001	0.002
AI_Feat_7	-0.014 ± 0.082	0.563 ± 0.391	0.278 ± 0.137	<0.001	<0.001	<0.001
AI_Feat_8	0.021 ± 0.085	0.606 ± 0.441	0.297 ± 0.138	<0.001	<0.001	0.001
AI_Feat_9	-0.023 ± 0.095	0.603 ± 0.404	0.324 ± 0.154	<0.001	<0.001	0.002
AI_Feat_10	-0.067 ± 0.017	-0.055 ± 0.029	-0.049 ± 0.019	0.073	0.014	0.608

AI_Feat_1 to AI_Feat_10 were extracted from the final baseline-trained 1D-CNN. Pairwise group comparisons were performed using the Mann–Whitney U test. These feature-level statistics were interpreted as exploratory latent-space analyses rather than independent diagnostic validation.

Supplementary Table S3. Exploratory apparent ROC analysis of PSV and final baseline-trained 1D-CNN-derived features.

Variable / model	Analysis type	Validation status	AUC
PSV only	Conventional scalar metric	Univariate ROC	0.695
Final baseline latent features	Exploratory latent-feature model	Apparent separability	0.994
Final baseline 1D-CNN probability	Exploratory final-model probability	Apparent separability	0.997

PSV was included as a conventional scalar reference. Latent-feature and final 1D-CNN probability analyses were derived from the final baseline-trained model and therefore represent apparent separability rather than independent validation.
